# Distributed Optical Fiber Strain Sensing for Monitoring Rainfall-Induced Deformation of Landslide-Prone Soil Slopes: A Physical Model Test

**DOI:** 10.3390/s26185701

**Published:** 2026-09-08

**Authors:** Lin Cheng, Jinliang Hu, Yang Cao, Xijia Liang, Xiao Han, Hao Peng, Junrui Chai, Chunhui Ma, Zhendong Yang

**Affiliations:** 1 State Key Laboratory of Water Engineering Ecology and Environment in Arid Area, Xi’an University of Technology, Xi’an 710048, China; hujl0225@163.com (J.H.); koleharper1436@gmail.com (X.L.); jrchai@xaut.edu.cn (J.C.); shanximachunhui@foxmail.com (C.M.); 2 Jilin Baishan Power Plant, Songhuajiang Hydropower Co., Ltd., Jilin 132012, China; xutcaoy@foxmail.com; 3 Power China Guiyang Engineering Corporation Limited, Guiyang 550081, China; hanxiao1124@sina.com (X.H.); penghaoslamdunk@yeah.net (H.P.)

**Keywords:** landslide deformation monitoring, distributed optical fiber strain sensing (DOFSS), optical fiber cable embedding technique, physical model test

## Abstract

To apply distributed optical fiber strain sensing (DOFSS) to the monitoring of rainfall-induced landslide deformation of landslide-prone soil slopes, this study proposes a deployment scheme and installation technique for strain-sensing optical fiber cables in soil slopes based on existing engineering experience and validates them through laboratory-scale physical model tests. The results show that direct burial of optical fiber cables in boreholes is a suitable installation method. A 2.0 mm tight-buffered optical fiber cable anchored with 5.0 mm heat-shrink-tube anchors was adopted as the fixed sensing configuration in the laboratory tests. In the laboratory model tests, the locations of strain peaks measured by the optical fiber cables were spatially associated with the visibly deformed regions, indicating that the strain anomalies can be used to approximately locate internal strain-concentration zones. The slope toe was identified as the location where localized failure was first observed, and the second rapid increase in strain at the slope toe may be regarded as a potential precursor of accelerated localized deformation under the tested condition. Borehole spacing has a significant influence on monitoring accuracy and is recommended to be controlled within 20–48% of the horizontal length of the potentially unstable slope zone. The variations in volumetric water content, earth pressure, and wetting front migration at different locations of the slope are highly correlated with rainfall infiltration and the evolution of slope surface erosion.

## 1. Introduction

Landslides are among the most frequent geological hazards and pose serious threats to human life and property. Previous studies have confirmed that rainfall is a key factor triggering slope instability and sliding [1], as it can significantly alter slope stability through multiple mechanisms, including increasing the self-weight of the soil mass, inducing surface erosion and scouring, and weakening soil shear strength. When the surface soil of a slope has a relatively high permeability coefficient, rainwater can readily infiltrate into the slope, leading to an increase in soil water content and the self-weight of the potential sliding mass, thereby reducing the overall stability of the slope. In contrast, when the surface soil has a low permeability coefficient or when rainfall intensity exceeds the infiltration capacity, rainwater cannot infiltrate rapidly and instead forms surface runoff along the slope surface. This runoff can strongly scour the slope surface, damage the surface structure, and cause soil loosening and detachment. In addition, infiltrated rainwater can increase pore water pressure within the slope, reduce effective stress, further weaken the shear strength of the soil, and substantially amplify the risk of slope failure. Landslide monitoring is a fundamental basis for investigating landslide formation mechanisms, prediction, and early warning, while continuous innovation in monitoring technologies is a key approach to improving landslide disaster prevention and mitigation.

Currently, commonly used landslide monitoring techniques include remote sensing (RS) [2], the Global Navigation Satellite System (GNSS) [3,4], and interferometric synthetic aperture radar (InSAR). These techniques can effectively capture surface deformation information of slopes; however, they are generally unable to characterize deep-seated displacement within slope bodies. For deep displacement monitoring, conventional methods mainly rely on inclinometers, settlement gauges, and multi-point extensometers [5,6,7], but their application is limited by insufficient capability for long-distance continuous monitoring. Distributed optical fiber strain sensing (DOFSS) has become an important technique for real-time monitoring, safety early warning, and operation management of geotechnical engineering structures because of its advantages in high-precision and long-distance deformation measurement. According to different sensing principles, DOFSS techniques mainly include optical time-domain reflectometry (OTDR), Brillouin optical time-domain reflectometry (BOTDR), Brillouin optical time-domain analysis (BOTDA), Brillouin optical frequency-domain analysis (BOFDA), and optical frequency-domain reflectometry (OFDR) [8]. Studies in the broader field of distributed fiber optic sensing indicate that monitoring performance may also be influenced by gauge length, spatial sampling, cable coupling, noise characteristics, and data-processing procedures [9]. In addition, cable structure and protective armoring may affect load-to-strain transfer and introduce nonlinear mechanical responses, suggesting that cable-specific characterization and calibration should be considered [10]. Owing to the irregularity and complexity of slope structures, higher requirements are imposed on the monitoring accuracy and reliability of DOFSS. Therefore, laboratory-scale model tests are often combined with field engineering tests to verify the feasibility of DOFSS in landslide monitoring and to systematically investigate the relevant influencing factors.

In terms of laboratory model tests, Li et al. [11] applied Brillouin optical time-domain reflectometry (BOTDR) and fiber Bragg grating (FBG) techniques to slope model tests, confirming the multiple advantages of the two optical fiber sensing techniques in landslide monitoring. Yan et al. [12] constructed an indoor soil slope model and used BOTDA technology to accurately capture the infiltration process and deformation characteristics of the slope under rainfall conditions, achieving precise identification of the sliding surface and surface deformation, which provides important data support for the study of slope failure mechanisms and prediction. Zhang et al. [13] adopted BOTDA technology and combined model tests with numerical simulation to study the deformation mechanism of slopes under cut-slope conditions. They found that the strain anomaly zones monitored by the optical fiber cable were highly consistent with the location of the potential sliding surface, and further analyzed the influence of rainfall and reservoir water level fluctuations on the measured cable strain. Leng et al. [1] used a distributed optical fiber sensing system to simultaneously monitor the internal temperature and strain of a loess slope model, revealing the evolution mechanism of a fractured loess slope under rainfall. Naruse et al. [14] successfully captured the dynamic strain variation during landslide occurrence through model tests with BOTDR technology. Suits et al. [15] systematically evaluated the performance of optical fiber monitoring data for slope failure early warning through flume model tests. Zhu et al. [16] built a medium-scale soil slope model in the laboratory and used BOTDA technology to monitor the horizontal strain distribution within the slope, verifying the capability of optical fiber sensors to effectively capture the deformation and failure process of the model slope. Zhu et al. [17] combined numerical simulation with laboratory model tests to establish an empirical relationship between the sensing cable strain and the landslide safety factor, showing greater practical convenience compared with traditional slope stability evaluation methods. Among the various distributed optical fiber sensing techniques, optical frequency-domain reflectometry (OFDR) can achieve mm-level spatial resolution and high-precision strain and temperature monitoring, which has significant advantages for application in laboratory-scale physical model tests [18,19].

In terms of landslide monitoring research in engineering practice, Shi et al. [20] explored the feasibility of BOTDR technology for landslide early warning. Ding et al. [21] designed an optical fiber sensing network based on BOTDR technology, achieving accurate analysis of abnormal zones and strain magnitudes in landslides. Sun et al. [22] applied DOFSS technology to the monitoring of the Majiagou landslide in the Three Gorges area of China, and various types of strain-sensing optical fiber cables effectively identified and located the surface deformation anomalies of the landslide mass, fully demonstrating the application advantages of DOFSS technology in practical landslide monitoring. Kogure et al. [23] adopted Rayleigh scattering-based distributed optical fiber sensing technology to conduct strain measurements along vertical profiles in landslide boreholes, successfully capturing earthquake-induced strain variations that could not be identified by traditional monitoring methods. Shi et al. [24] appropriately deployed sensing optical fiber cables in an embankment project, effectively capturing the location and extent of abnormal deformation of the embankment slope, which provided a scientific basis for engineering treatment decisions. Zhu et al. [25] investigated the correlations among the displacement at the loading point, the cable sliding distance, and the optical loss through laboratory single-pile tests using OTDR technology, confirming the application potential of optical fiber cables in landslide monitoring. In their study of landslide early-warning systems, Yin [26,27] focused on the successful application of BOTDR technology in the monitoring of the Wushan New Town landslide in China, and proposed that the distributed optical fiber sensing system could be extended to the monitoring of the remaining 3200 landslides in the Three Gorges Reservoir area. Sun et al. [28] constructed a multi-field monitoring system for landslides based on BOTDR technology, achieving the synchronous monitoring of strain, temperature, and seepage, and successfully applied it to the Majiagou landslide. Xu et al. [29] verified through field tests that BOTDA technology can effectively identify the strain and stress distribution characteristics along the anchor rods in reinforced slopes.

In engineering practice, key issues such as the burial position, layout spacing, and type selection of sensing optical fiber cables are mostly determined based on field experience, and unified design specifications and deployment standards remain limited. Consequently, the repeatability and applicability of monitoring results under different site conditions may be limited, making it difficult to fully leverage the advantages of optical fiber sensing technology in terms of measurement accuracy and stability for geological hazard early warning. Compared with many previous studies that primarily demonstrated the feasibility of DOFSS for monitoring slope deformation, this study focuses on the design and evaluation of the deployment process. The methodological contribution lies in integrating the numerical estimation of the potential deformation range, borehole-based cable layout, a sequential installation procedure, and laboratory evaluation of deployment direction and borehole spacing into a deployment-oriented monitoring framework.

In this study, a homogeneous loess slope with a height of 25 m was selected as the prototype for designing the laboratory model to investigate a distributed optical fiber monitoring approach for rainfall-induced deformation of landslide-prone soil slopes. First, the deployment scheme and installation procedure for the distributed optical fiber sensing network were designed, specifying the cable layout method, borehole spacing, borehole depth, cable type selection, and sequential construction workflow. Then, using the loess slope as the prototype, laboratory-scale model tests were conducted under different rainfall intensities and cable deployment conditions. Finally, the wetting front, moisture content, earth pressure, and measured strain data from the optical fiber cables were analyzed to summarize the monitoring characteristics, evaluate the effects of different deployment conditions, and identify potential deformation precursors.

## 2. Design and Construction of a Distributed Optical Fiber Sensing Network for Soil Slopes

**Figure 1:**
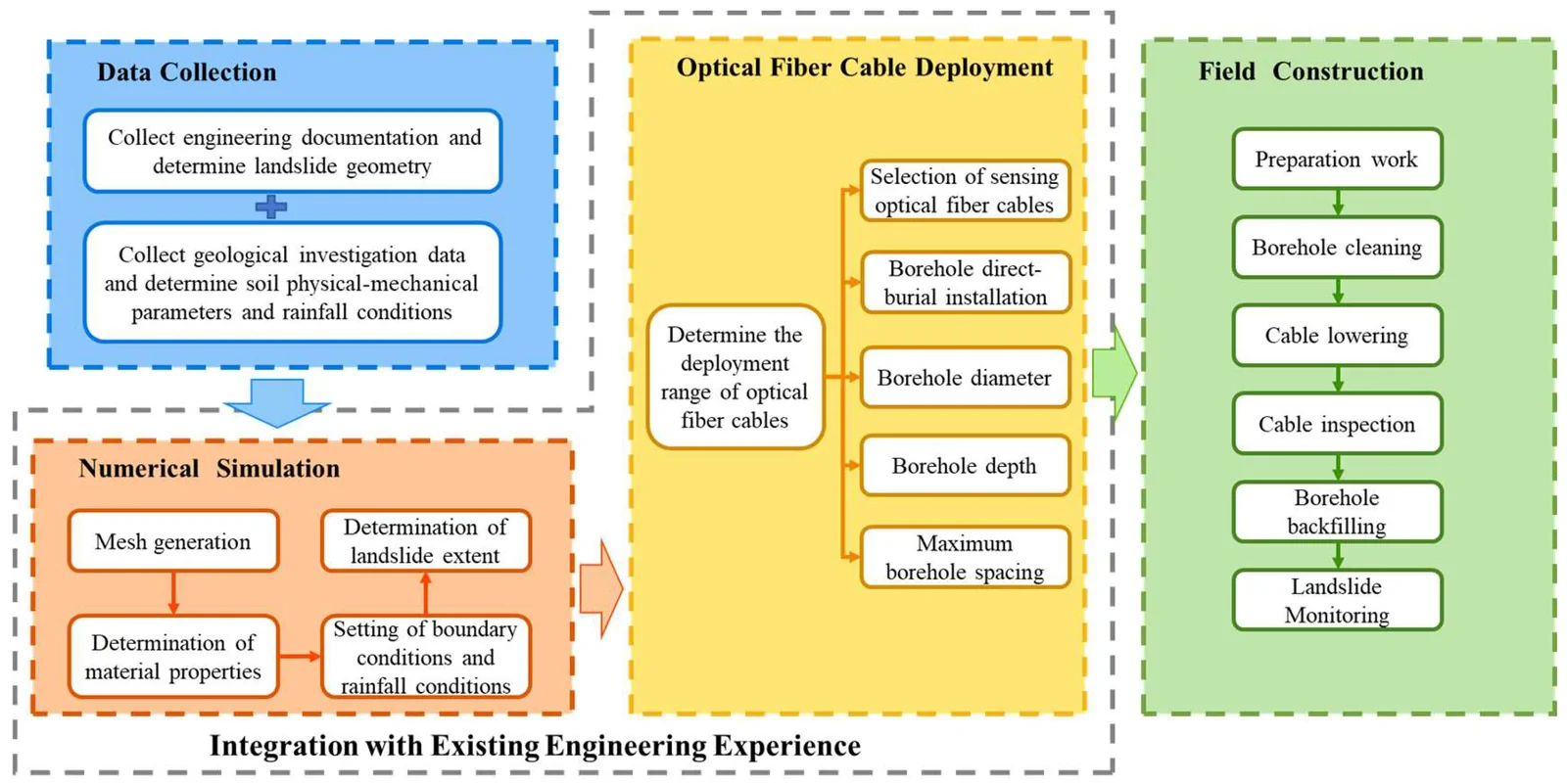
Deployment scheme and construction procedure for strain-sensing optical fiber cables in practical engineering soil slopes.

With reference to relevant literature [30,31] and existing engineering experience, this study develops a deployment-oriented scheme and construction procedure for distributed optical fiber cables in practical soil slopes, as shown in Figure 1. The framework links engineering data collection, numerical estimation of the potential deformation range, cable-layout design, and borehole construction into a sequential workflow for slope monitoring.

### 2.1. Design of the Optical Fiber Cable Deployment Scheme

(1) Deployment Method for Sensing Optical Fiber Cables

According to the contact relationship between the sensing optical fiber cable and the soil mass, cable deployment methods can be divided into two types: direct burial and attached installation. The attached installation method involves bonding the optical fiber cable to artificial structures, such as inclinometer casings and anti-slide piles. In this way, the strain of the landslide mass is indirectly reflected by the strain of the host structure to which the cable is attached. At present, the direct-burial method is more widely used. In this method, the sensing optical fiber cable is directly placed into the soil mass through trenching or borehole drilling. If deep-seated deformation of the landslide is to be monitored, the cable should be embedded through borehole drilling.

(2) Borehole Spacing and Borehole Depth

To monitor a landslide, the engineering geological investigation report of the slope should first be collected to clarify the stratum distribution, groundwater level, and the location and depth of the potential sliding surface, with the aim of identifying as many potential slip surfaces in the deep part of the landslide mass as possible. For homogeneous soil slopes, the finite element method can be adopted to analyze the deformation response and potential sliding range of the slope induced by rainfall and other factors, and to calculate the location and shape of the sliding surface. For homogeneous cohesive soil, the sliding surface is generally arc-shaped. Based on the numerical simulation results, the spatial distribution of the landslide toe, rear scarp elevation, and overall sliding surface can be determined, thereby providing a key basis for determining the borehole depth and horizontal deployment range of the monitoring system.

To comprehensively capture the deformation characteristics within the landslide mass, optical fiber cables need to be deployed in the front, middle, and rear sections of the landslide body, with at least one borehole in each section. Table 1 summarizes the borehole spacing of some existing optical fiber monitoring schemes for landslides. As shown in Table 1, the ratio of borehole spacing to the horizontal projection length of the landslide ranges from 0.20 to 0.48. Based on this empirical data, the maximum horizontal distance between monitoring boreholes is set to one-third of the horizontal projection length of the landslide body. In general, the borehole depth should penetrate all possible slip surfaces with a certain margin, so as to ensure that all potential large-deformation zones can be captured.

(3) Selection of Sensing Optical Fiber Cable Type

In landslide monitoring, since landslide areas typically undergo significant deformation, the proper selection of the sensing optical fiber cable type is key to extending the service life of the monitoring system. At present, tight-buffered strain-sensing optical fiber cables are the primary choice for deformation monitoring in practical engineering structures. A tight-buffered sensing cable is generally composed of a fiber core, cladding, protective coating, and outer sheath. It has greater mechanical robustness, can accommodate a larger deformation range, is less prone to fiber breakage, and is therefore more suitable for field construction and long-term deployment.

(4) Selection of Sensing Technology

The selection of distributed optical fiber sensing technology should be made based on a comprehensive consideration of factors such as the monitoring objectives, the scale of the landslide mass, the length of cable deployment, the spatial resolution requirements, the measurement accuracy, the field access conditions, installation and maintenance requirements, and overall implementation cost. For practical engineering slopes, the monitored objects are typically characterized by large scale, wide spatial extent, and long monitoring duration. Therefore, the sensing technology should primarily satisfy the requirements of long-distance continuous monitoring, stable data acquisition, and adaptability to field construction conditions. Different distributed optical fiber sensing technologies differ in their sensing mechanisms, spatial resolution, monitoring distance, implementation complexity, access methods, and applicable scenarios. A comparison of their main technical characteristics and applicability is presented in Table 2.

As shown in Table 2, OTDR, BOTDR, and BOTDA offer advantages in monitoring distance and field adaptability, making them more suitable for large-scale, long-distance continuous monitoring in practical engineering slopes. OTDR technology is primarily applicable for identifying distributed disturbances, optical loss variations, and macro-scale deformation responses. BOTDR technology features single-end access and imposes lower requirements on cable loop integrity, making it suitable for engineering scenarios with complex construction conditions or limited access at the distal end. BOTDA technology provides a higher signal-to-noise ratio and measurement accuracy, and is suitable for monitoring scenarios where both ends of the cable are accessible and high accuracy of distributed strain measurement is required. In comparison, OFDR technology offers higher spatial resolution and can obtain high-density distributed strain information over short distances, making it suitable for laboratory model tests and fine-scale identification of localized deformation concentration zones.

In this study, the laboratory model test has a relatively small spatial scale, a limited range of slope deformation development, and requires the identification of local strain anomalies near the slip potential deformation zone. Spatial resolution is therefore a key factor affecting the monitoring performance. Consequently, this study adopts OFDR technology as the distributed strain demodulation scheme for the laboratory model test, so as to improve the characterization of the deformation process and localized strain-concentration zones in the small-scale slope model. For field-scale slopes, the demodulation technology should be selected according to the required monitoring distance and spatial resolution. BOTDR or BOTDA can be used for long-distance coverage, whereas OFDR may be applied to localized key zones requiring finer spatial resolution. The borehole-based installation procedure and the observed effects of deployment direction and spacing may provide a reference for designing such field monitoring layouts.

### 2.2. Embedding Procedure of Sensing Optical Fiber Cables

The procedure for embedding sensing optical fiber cables through borehole drilling in a landslide mass is as follows:

(1) Preparation Work

First, the sensing optical fiber cable is calibrated through a calibration test to measure the relevant sensing parameters, and the results are compared with the factory values to ensure that the cable is not damaged. The middle section of the sensing cable is wound around a guide weight. The guide weight is selected according to the borehole depth and diameter, and the binding point at the head of the guide weight is protected using materials such as heat-shrink tubes.

(2) Borehole Cleaning

After the borehole drilling is completed, the loose soil debris remaining in the borehole needs to be preliminarily cleaned. For loose soil structures such as loess and silt, flushing with a large amount of clean water should be avoided to prevent borehole wall collapse or disturbance of the in situ soil structure. Gravity sedimentation, slag extractors, or other gentle methods can be used to remove the sediment at the bottom of the borehole. After cleaning, the cable lead-in section is threaded through the guide weight, and the cable is firmly connected to the guide weight to form a complete loop, in preparation for lowering and embedding. It should be noted that the so-called “cable lead-in section” does not refer to a separate “wire” or “control line,” but rather to the portion of the original cable that is not used for sensing; it connects the demodulator to the sensing section to complete the data loop.

(3) Lowering of the Sensing Optical Fiber Cable

First, the guide weight with the fixed cable and lead-in section is lowered. Then, the drill rod is placed into the tail sleeve of the guide weight, and finally, the cable is carried to the deep part of the borehole by lowering the drill rod. During lowering, any external force on the cable should be avoided. Therefore, only the steel wire rope fixed to the cable should be pulled, and while lowering the cable, the lead-in section is tied to the steel wire rope. Typically, a portion of the lead-in section is tied every 2–3 m. This tying operation prevents the lead-in section from sliding or kinking, thereby ensuring smooth cable lowering.

(4) Re-Calibration of the Sensing Optical Fiber Cable

After the cable has been lowered to the bottom of the borehole, the cable is tested again using the instrument to ensure that it has not been damaged during the series of operations. To achieve a better compression measurement effect of the cable, the cable is pulled upward, and the tension is adjusted based on the detected strain. During the entire process, the cable should be kept as straight as possible.

(5) Tensioning of the Sensing Optical Fiber Cable

After the cable has been fixed in place, the upper end of the lead-in section is tied to a post near the borehole to keep it in a tensioned state, so as to prevent the cable from sliding downward.

(6) Borehole Backfilling

Appropriate backfill material is selected for borehole backfilling based on the soil conditions around the borehole. During the backfilling process, the backfill material should be poured slowly to avoid damage to the cable and to ensure that the backfill is compact. The sensing optical fiber cable should be kept in a straight state throughout the entire process. At present, sand-clay mixtures are commonly used as backfill material for boreholes in optical fiber-based landslide monitoring projects, and some projects use gravel and medium-coarse sand. However, excessive particle size may lead to insufficient compaction and thus affect the measurement accuracy. Jiang Na [30] confirmed that, for sand-clay mixed backfill, the higher the clay content, the better the cable–soil coupling performance. Meanwhile, Shi Songge [31] experimentally verified that the cable–sand coupling performance decreases with increasing sand particle size. For loess landslides, since loess is a cohesive soil and cohesive soil has a positive effect on cable–soil coupling, loess can be directly used as backfill material. Although cohesive backfill can improve cable–soil coupling, the measured cable strain should be interpreted as a response transferred through the cable–soil interface rather than as an exact measure of soil strain. Local debonding, cable slippage, and time-dependent changes in the interface may reduce strain-transfer efficiency, particularly under large deformation or long-term field conditions.

(7) Strain Data Acquisition

After the backfill material has stabilized, the demodulation equipment is used to obtain the strain distribution at the corresponding positions in the borehole. Once the sensing optical fiber cable has been embedded, connecting the demodulation equipment for data acquisition enables the distributed monitoring of deep-seated deformation within the landslide mass.

## 3. Experimental Evaluation of the Optical Fiber Cable Deployment Method

To evaluate the proposed deployment scheme for sensing optical fiber cables and analyze the deformation evolution of loess slopes under rainfall conditions, a scaled slope model was established through laboratory model tests. A rainfall simulator was used to simulate different rainfall conditions, and a multi-parameter monitoring system equipped with various types of sensors was deployed to simultaneously record the dynamic responses of the slope under rainfall conditions, thereby revealing its deformation evolution characteristics.

### 3.1. Experimental System

**Figure 2:**
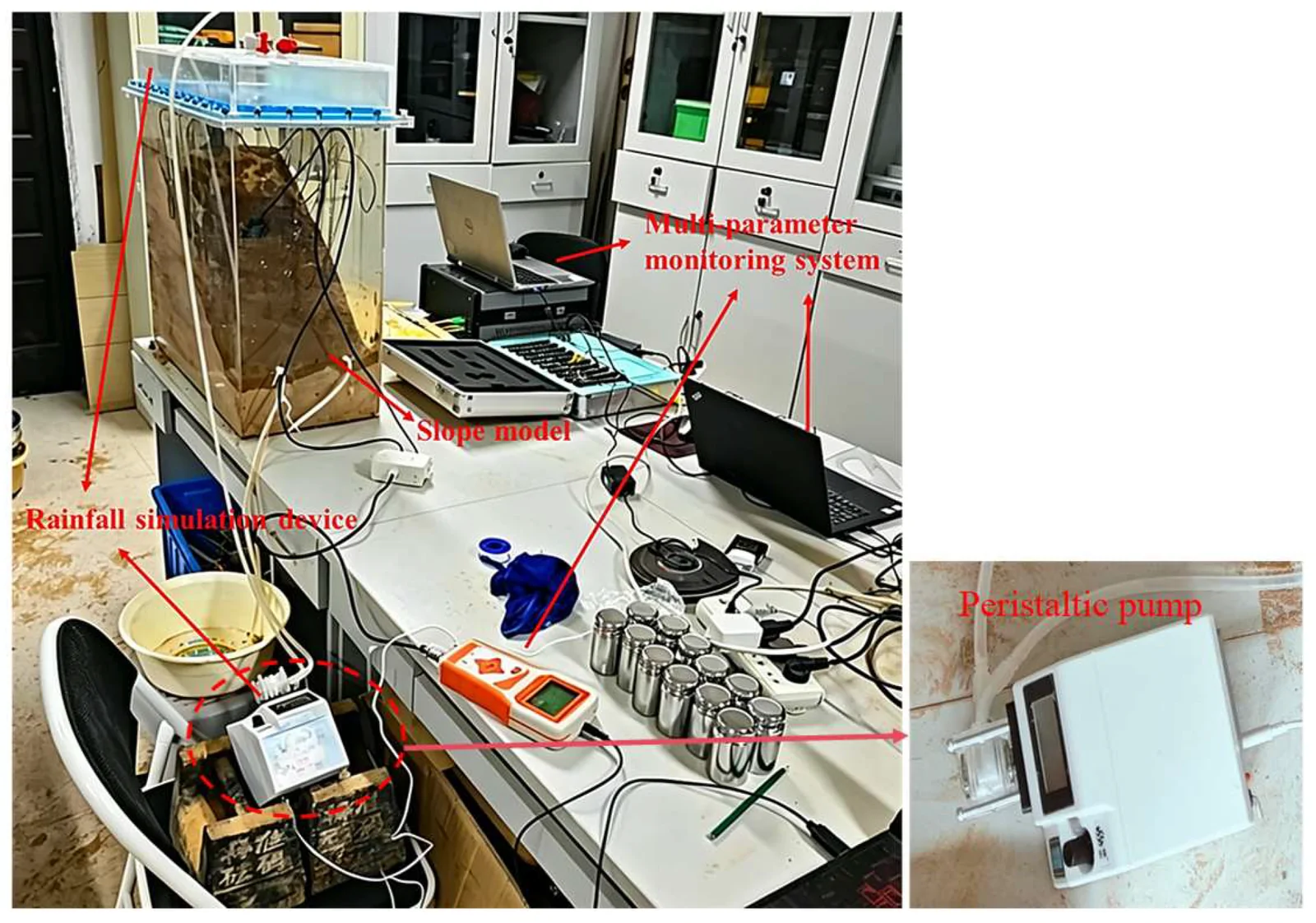
Photograph of the experimental system setup.

As shown in Figure 2, the experimental system mainly consists of three parts: the scaled slope model, the rainfall simulation device, and the multi-parameter monitoring system.

#### 3.1.1. Scaled Slope Model

A scaled laboratory slope model was designed based on a prototype homogeneous loess slope with a height of 25.0 m located in the Heifangtai area of Gansu Province, China. The prototype loess slope has a slope angle of 45°, classifying it as a steep slope with a high risk of landslides and collapses. The similarity ratios for soil density, slope geometry, and gravitational acceleration were taken as *C_ρ_* = 1, *C_l_* = 50 and *C_g_* = 1, respectively. The test soil was collected from the prototype site. After remolding, the soil parameters exhibited only slight variation; therefore, the similarity ratios for physical parameters such as density, cohesion, shear modulus, elastic modulus, and friction angle of the loess were all taken as unity. Based on the Buckingham π theorem and dimensional analysis, the physical quantities in the test can be expressed as:

(1) $$f=\left(\pi_{1},\pi_{2},\pi_{3},\pi_{4},\pi_{5},\pi_{6},\pi_{7},\pi_{8},\pi_{9},\pi_{10}\right)$$

(2) $$\pi_{1}=\frac{d}{l}$$

(3) $$\pi_{2}=\frac{tg^{1/2}}{l^{1/2}}$$

(4) $$\pi_{3}=\frac{F}{l\rho g}$$

(5) $$\pi_{4}=\frac{c}{l\rho g}$$

(6) $$\pi_{5}=\frac{G}{l\rho g}$$

(7) $$\pi_{6}=\varphi$$

(8) $$\pi_{7}=v$$

(9) $$\pi_{8}=\frac{\sigma }{l\rho g}$$

(10) $$\pi_{9}=\frac{E}{l\rho g}$$

(11) $$\pi_{10}=\varepsilon$$

where *l* is the geometric length, *d* is the displacement, *t* is the time, *c* is the cohesion, *φ* is the internal friction angle, *g* is the gravitational acceleration, *ρ* is the density, *E* is the elastic modulus, *G* is the shear modulus, *υ* is Poisson’s ratio, *σ* is the stress, and *ε* is the strain. The similarity ratios of these parameters are shown in Table 3.

During the sampling process, the original natural structure of the soil was significantly disturbed, and the soil structure was essentially lost. Therefore, the soil sample was remolded according to the density measured in the laboratory before the test, and the basic physical parameters of the loess were determined through tests, as shown in Table 4.

**Figure 3:**
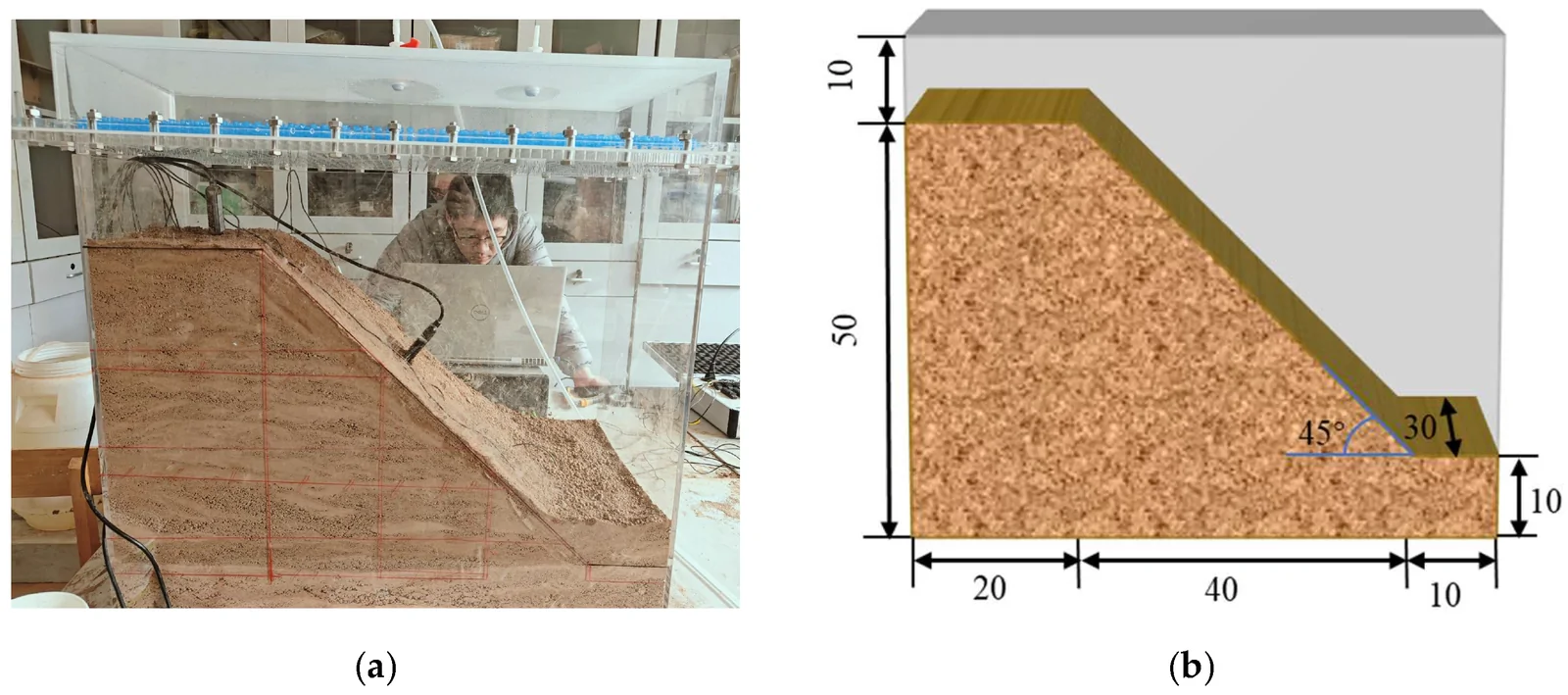
Scaled slope model: (**a**) Photograph of the scaled slope model; (**b**) model dimensions (unit: cm).

The scaled slope model was placed in a transparent test box, as shown in Figure 3. The test box had dimensions of 70 cm × 30 cm × 60 cm (length × width × height). The transparent rigid walls facilitated visual observation of the deformation process while providing lateral confinement to the model. Four drainage outlets with a diameter of 1 cm were arranged on the front and rear sides of the box, 5 cm above the bottom, to control drainage near the base. Thus, the observed seepage and deformation responses were obtained under the prescribed lateral-confinement and drainage conditions.

#### 3.1.2. Rainfall Simulation Device

**Figure 4:**
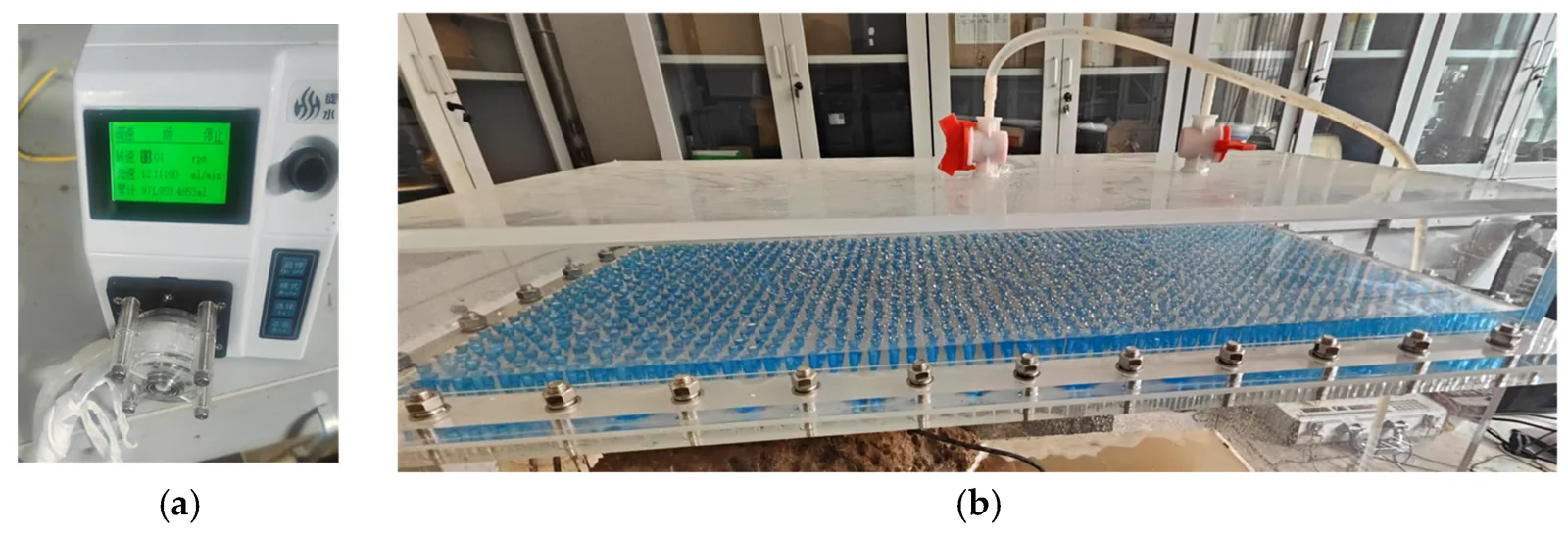
Rainfall simulation device: (**a**) Digital display interface of the peristaltic pump; (**b**) rainfall plate.

The rainfall simulation system consists of a water storage tank, a peristaltic pump, PVC hoses, a water control valve, and a rainfall plate. The rainfall simulation device connects the components through rubber hoses, and uniform rainfall under different rainfall intensities can be simulated by controlling the pumping power of the peristaltic pump. A 16 L plastic water storage tank is used as the water source, and water is supplied by the peristaltic pump. To maintain a constant water pressure, the water level in the tank should be kept stable, thereby ensuring that the rainfall effect meets the experimental requirements regardless of power fluctuations of the pump. The maximum flow rate of the peristaltic pump is 550 mL/min, which facilitates flow regulation under different rainfall intensities. The digital display of the peristaltic pump can record the total water consumption at the end of the test, as shown in Figure 4a. PVC hoses are used to deliver water from the storage tank to the peristaltic pump and the rainfall plate. A water control valve is installed on the hose segment connected to the rainfall plate, which can precisely regulate the flow velocity and flow rate of the water, thereby ensuring that the intensity and coverage of the artificial rainfall meet the experimental design requirements. Water is delivered from the peristaltic pump to the rainfall plate. The rainfall plate consists of an upper water storage groove and a lower pinhole nozzle array, which can uniformly disperse water to the required area, simulating the distribution pattern of natural raindrops, as shown in Figure 4b.

By controlling the flow rate of the peristaltic pump, different rainfall intensities can be achieved. The relationship between rainfall intensity and the flow rate of the peristaltic pump can be converted using the following equation:

(12) q=60Q/S

where *q* is the rainfall intensity (mm/h), *Q* is the flow rate of the peristaltic pump (L/min), and *S* is the effective rainfall area (m^2^). In this test, the effective area of the rainfall plate was 0.21 m^2^.

#### 3.1.3. Multi-Parameter Monitoring System

The multi-parameter monitoring system consisted of four components: soil moisture content monitoring, earth pressure monitoring, distributed optical fiber strain monitoring, and high-speed camera video monitoring. All sensors were embedded in the middle cross-section of the test model.

(1) Soil Moisture Content Monitoring

**Figure 5:**
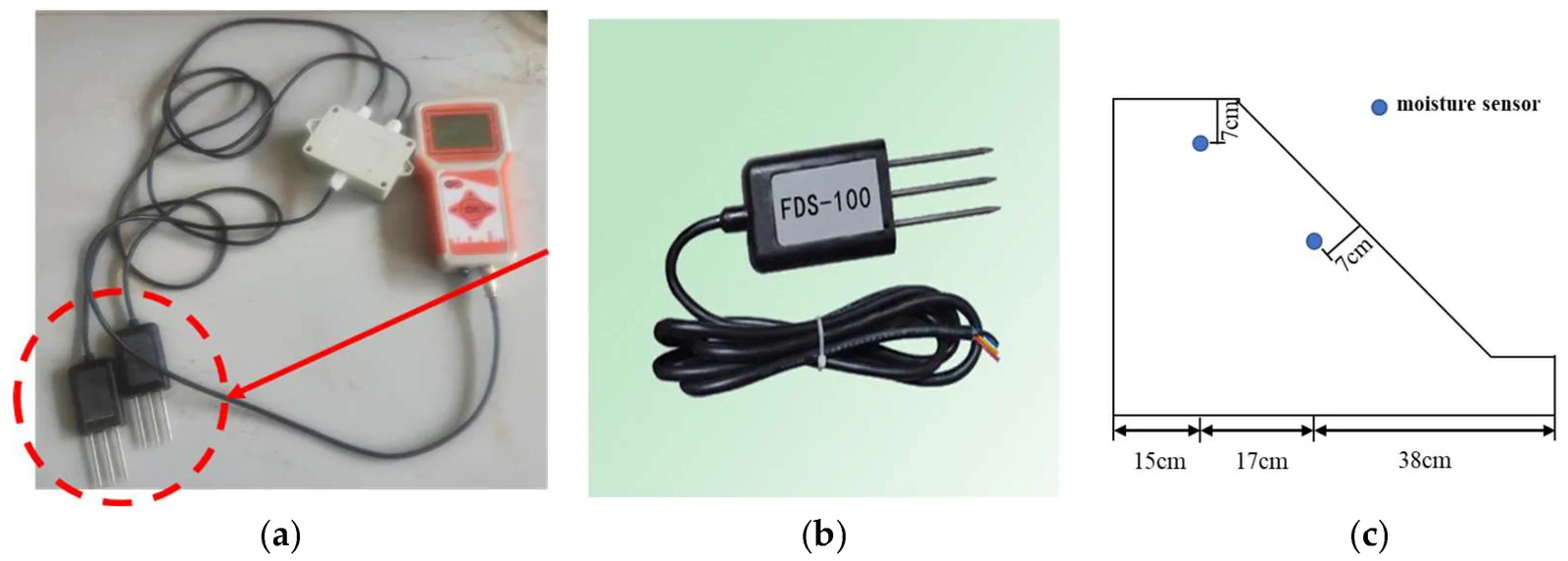
Soil moisture content monitoring system: (**a**) Multi-parameter soil rapid tester; (**b**) moisture sensor; (**c**) sensor layout (unit: cm).

As shown in Figure 5, two FDS-100 soil moisture content sensors (produced by Qingyi Electronics (Tianjin) Co., Ltd., Tianjin, China.) were used in this test to monitor the moisture content of the slope soil. The sensors were connected to a multi-parameter soil rapid tester for real-time monitoring of the soil moisture content during the rainfall infiltration process. The soil moisture sensor is based on the frequency domain reflectometry (FDR) method. It utilizes the electromagnetic pulse principle to measure the apparent dielectric constant (*ε*) of the soil according to the propagation frequency of electromagnetic waves in the medium, thereby obtaining the volumetric water content (*θ_v_*) of the soil. The measurement accuracy is ±1%, the measurement range is 0–100%, and the resolution is 0.1%. The measurement volume is a cylinder with the central probe as the center, having a diameter of 7 cm and a height of 7 cm.

(2) Earth Pressure Monitoring

**Figure 6:**
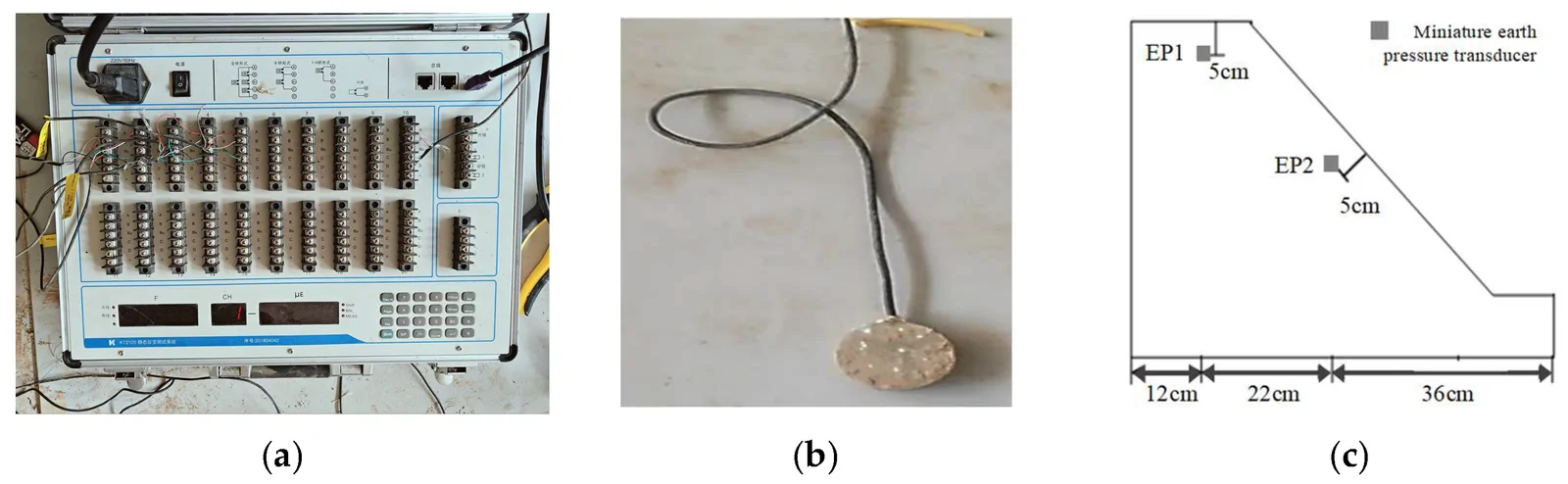
Earth pressure monitoring system: (**a**) Static strain test system; (**b**) miniature earth pressure transducer; (**c**) locations of EP1 and EP2 in the slope model (unit: cm).

As shown in Figure 6 two miniature earth pressure transducers, denoted EP1 and EP2, were embedded at the slope crest platform and mid-slope, respectively, at a burial depth of 5 cm below the local slope surface. The miniature earth pressure transducers had a measurement range of 0–1.0 kPa, an allowable overload of 120% FS, and an error of ±1.0% FS. A KT2120 static strain test system was connected to the miniature earth pressure transducers for data acquisition to record the earth pressure data.

(3) Distributed Optical Fiber Strain Monitoring

For strain monitoring in this test, a tight-buffered single-mode optical fiber cable with an outer diameter of 2 mm (model NZS-DSS-C07) was selected. An OSI-S OFDR high-precision distributed optical fiber demodulator (sourced from Suzhou NanZee Sensing Technology Co., Ltd. (Nanzhi Sensing), Suzhou, Jiangsu Province, China) was used for data acquisition, with a nominal strain accuracy of 1 με, a measurement range of −20,000 με to +20,000 με, and a maximum spatial resolution of 1 mm. The spatial resolution used in the test was 1 cm. To enhance the coupling between the strain-sensing optical fiber cable and the soil, a heat-shrink tube with a diameter of 5.0 mm was selected to anchor the cable, based on a balance between economic efficiency and mechanical performance. The heat-shrink tube is easy to install, and the cylindrical anchoring configuration provides a larger contact area with the soil. The anchoring spacing was set at 20 cm, following the study of Zhang [13]. The 2 mm cable diameter, 5 mm heat-shrink tube diameter, and 20 cm anchoring spacing were maintained consistently in all anchored test conditions.

(4) High-Speed Camera Video Monitoring

A dual high-speed camera system was used for full-process recording in this test. The main camera was fixed on the side of the transparent plexiglass to capture the macroscopic deformation processes, such as water infiltration and slope body displacement. The auxiliary camera was handheld to dynamically track the evolution of surface runoff, the erosion process, and local deformation details. This dual-camera configuration enabled multi-angle synchronous observation of the slope failure process, ensuring both the continuous recording of the overall deformation field and the accurate capture of the evolution characteristics of key zones.

### 3.2. Estimation of Potential Sliding Surface Based on Numerical Analysis

**Figure 7:**
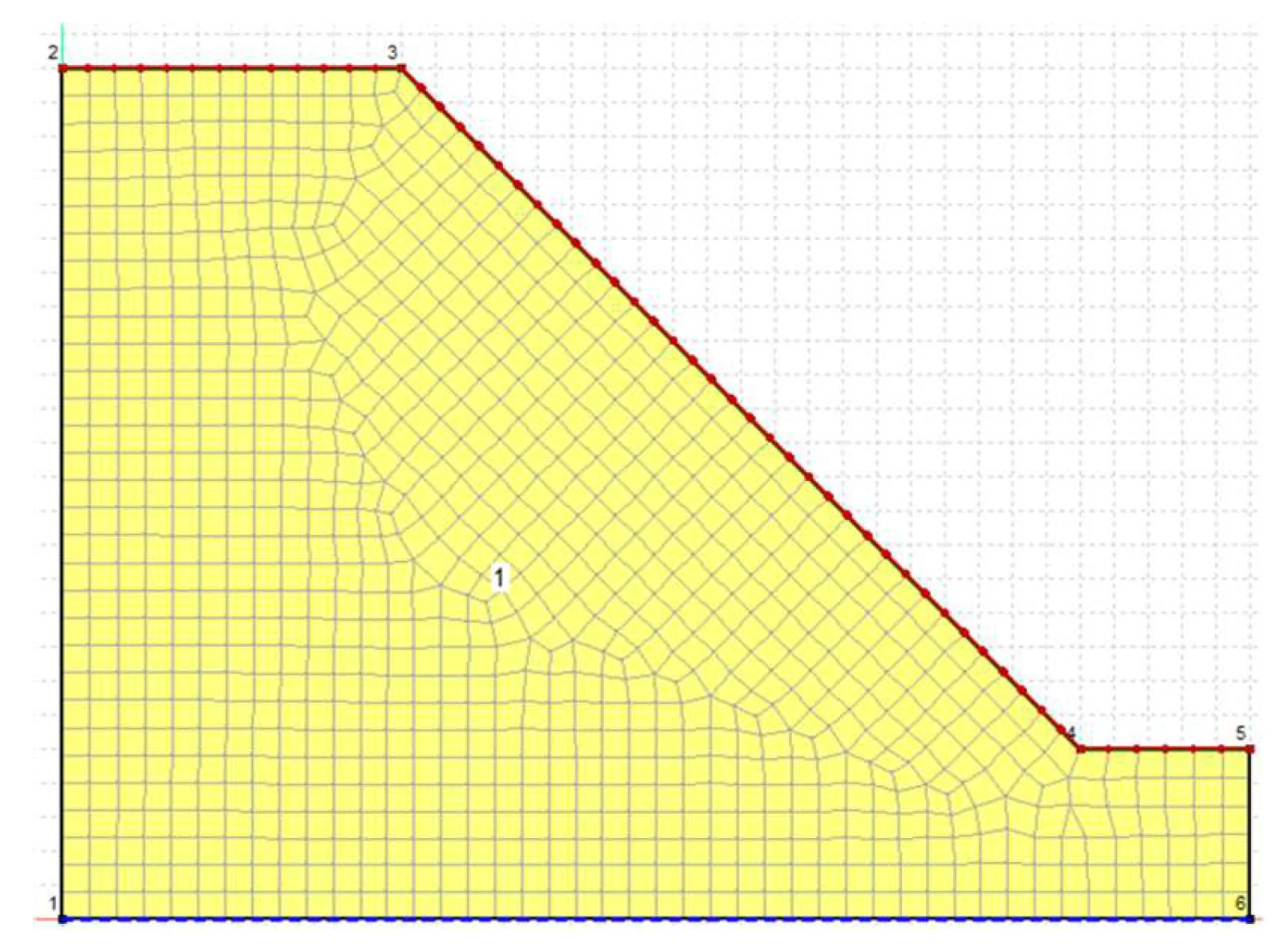
Numerical model and mesh of the prototype loess slope.

In this study, the seepage and stability analysis modules of the Geo-Studio software(Version 2021.4) were used to analyze the extent of the slip surface in the slope under heavy rainfall conditions. The potential landslide range corresponding to the minimum safety factor was taken as the key area for deployment of the strain-sensing optical fiber cables. A computational model was established based on the prototype loess slope, and a mixed mesh of quadrilateral and triangular elements was adopted for mesh generation. The computational model consisted of 968 nodes and 901 elements, as shown in Figure 7.

**Figure 8:**
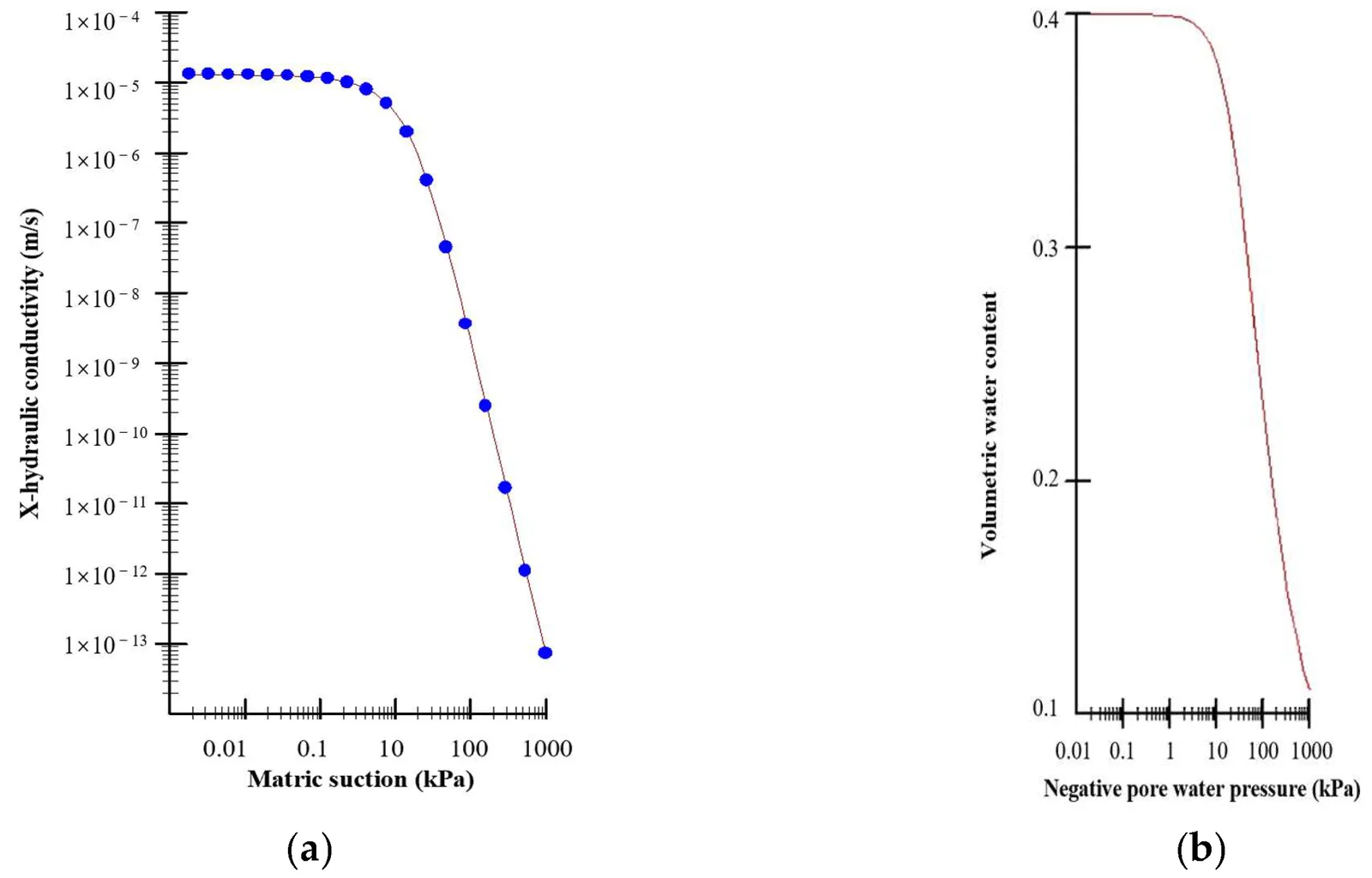
Permeability function and volumetric water content curve: (**a**) Permeability function; (**b**) volumetric water content curve.

The seepage analysis adopted a saturated–unsaturated seepage model. The saturated volumetric water content and saturated hydraulic conductivity used in the model were obtained from laboratory measurements of the loess. The SWCC was represented using the van Genuchten model, a was taken as 30, n as 1.5, m as 0.33, a saturated volumetric water content of 40%, and a residual volumetric water content of 5%. Based on the adopted SWCC and saturated hydraulic conductivity, the permeability function was estimated using the built-in prediction method in SEEP/W. No inverse calibration against the physical-model observations was performed. The permeability function and the soil–water characteristic curve are shown in Figure 8.

Among the empirical formulas for unsaturated soil fitting, the soil–water characteristic curve model proposed by van Genuchten has the advantages of a wide suction range and a fitted curve that is closer to the actual SWCC. Therefore, it has been adopted by most researchers [40,41,42]. Based on the soil–water characteristic curve, van Genuchten proposed the following power-law fitting expression for the relationship between volumetric water content and matric suction in unsaturated soil:

(13) $$\theta_{w}=\theta_{r}+\frac{\theta_{s}-\theta_{r}}{{\left[1+{\left(\frac{s}{a}\right)}^{n}\right]}^{m}}$$

where $\theta_{w}$ is the volumetric water content at the calculation time step, $\theta_{s}$ and $\theta_{r}$ are the saturated and residual volumetric water contents, respectively, *a* is the soil parameter related to the air-entry value function, s is the pressure head, and n and m are the fitting parameters.

According to the rainfall intensity standards specified by the meteorological department, heavy rainfall conditions were selected to determine the sliding surface location. The rainfall intensity was set at 100 mm/d, and the rainfall duration was 12 h. A zero-head boundary was set at the bottom of the model as a far-field hydraulic boundary condition to approximate the seepage field simulation.

**Figure 9:**
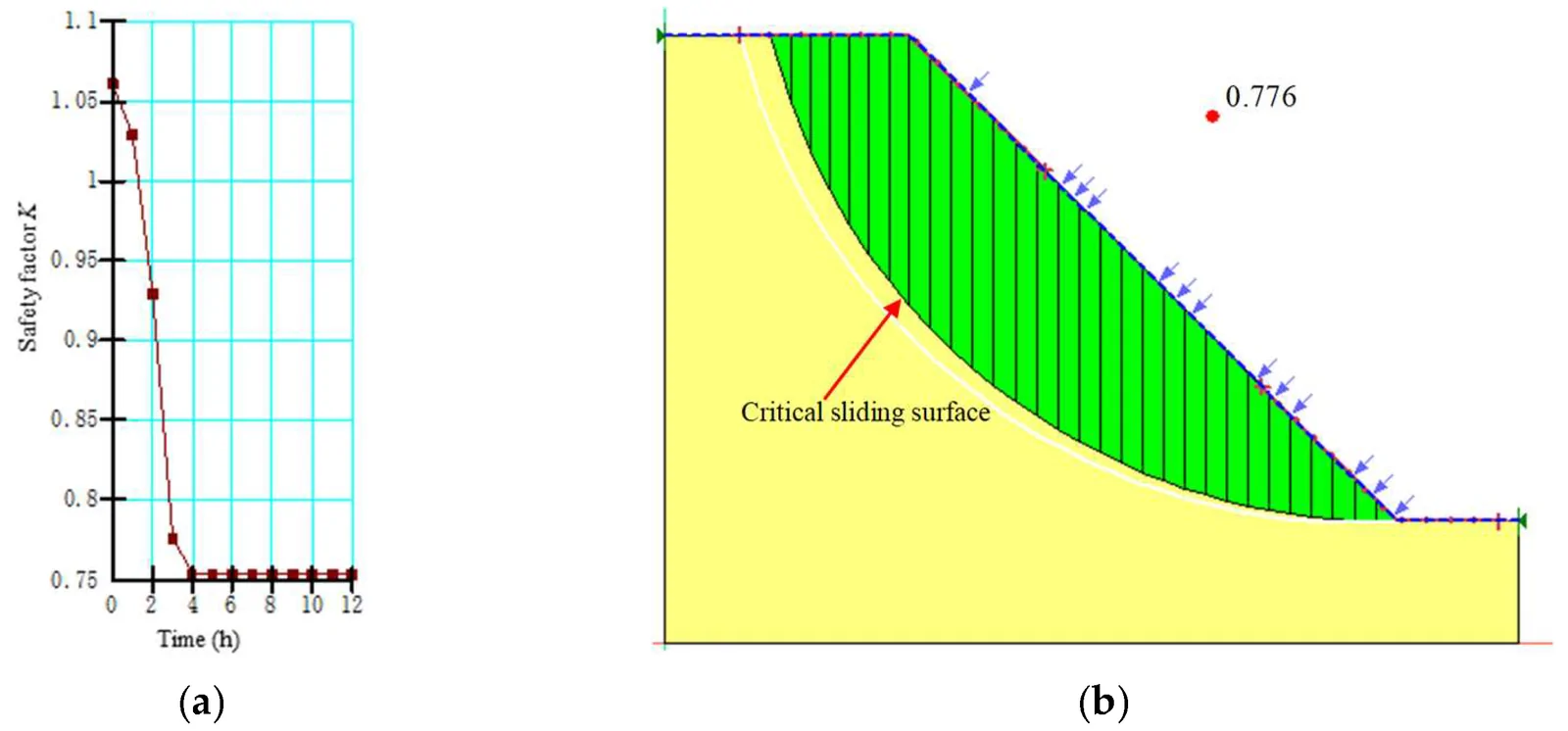
Numerical simulation results: (**a**) Variation of safety factor with time; (**b**) potential sliding surface corresponding to the minimum safety factor.

In accordance with the Specification for Design and Construction of Landslide Control Engineering (DZ/T 0219-2006) [43], the anti-sliding stability safety factor of slope control engineering under the combined effects of self-weight, heavy rainfall, and groundwater should satisfy K = 1.02–1.15. The calculation results showed that the initial safety factor of the slope was 1.06, which was within the stable range. The safety factor decreased with rainfall duration and stabilized after 4 h, as shown in Figure 9a. Accordingly, the potential sliding surface corresponding to the minimum safety factor was adopted as the basis for optical fiber cable deployment. As shown in Figure 9b, the most critical potential sliding surface extended from a position 4.0 m below the slope crest platform to the slope toe, with a horizontal length of 26 m. The predicted potential sliding surface was used to determine the horizontal deployment range and borehole depth of the optical fiber monitoring system.

### 3.3. Test Conditions

Three rainfall conditions, namely rainstorm, moderate rain, and light rain, were set in this test to investigate the internal response mechanism of the slope under different rainfall intensities. The rainfall intensities corresponding to the three conditions were determined according to the Grade of Precipitation (GB/T 28592-2012) [44], as shown in Table 5.

**Figure 10:**
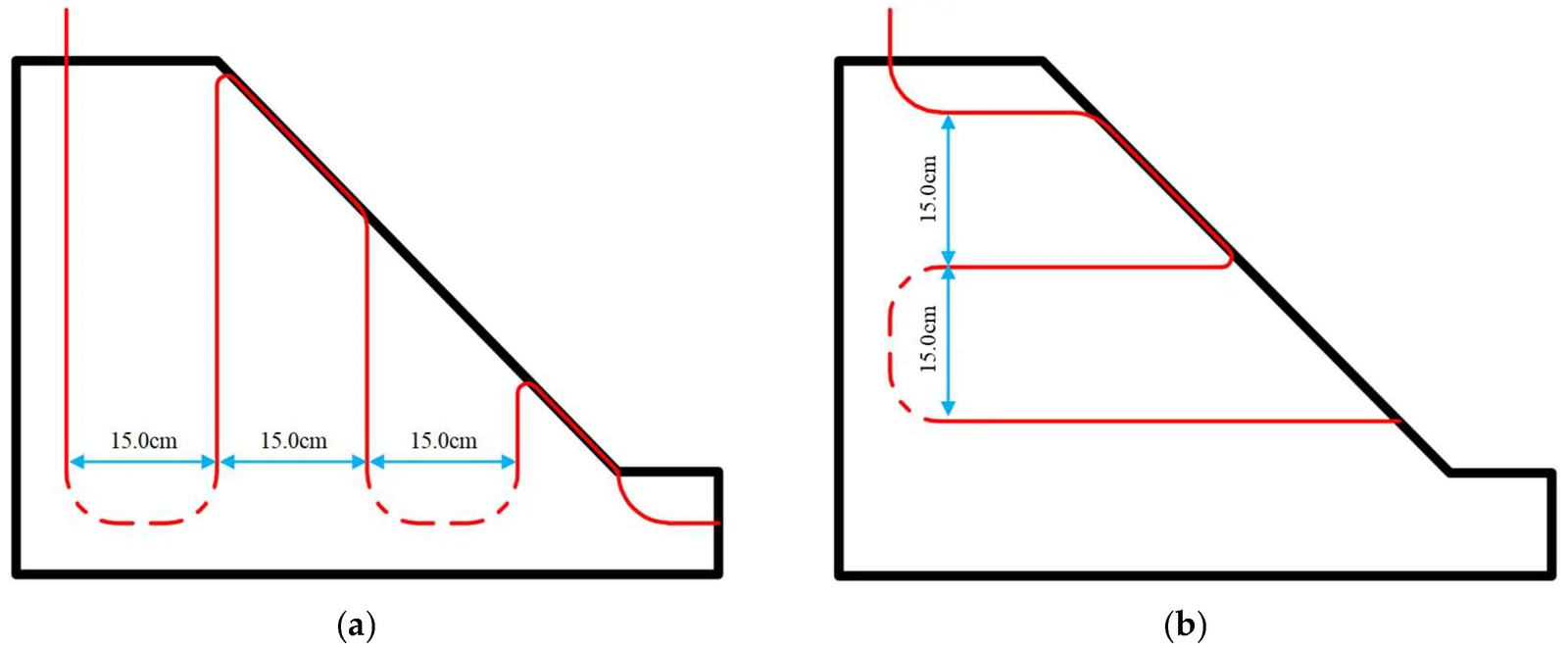

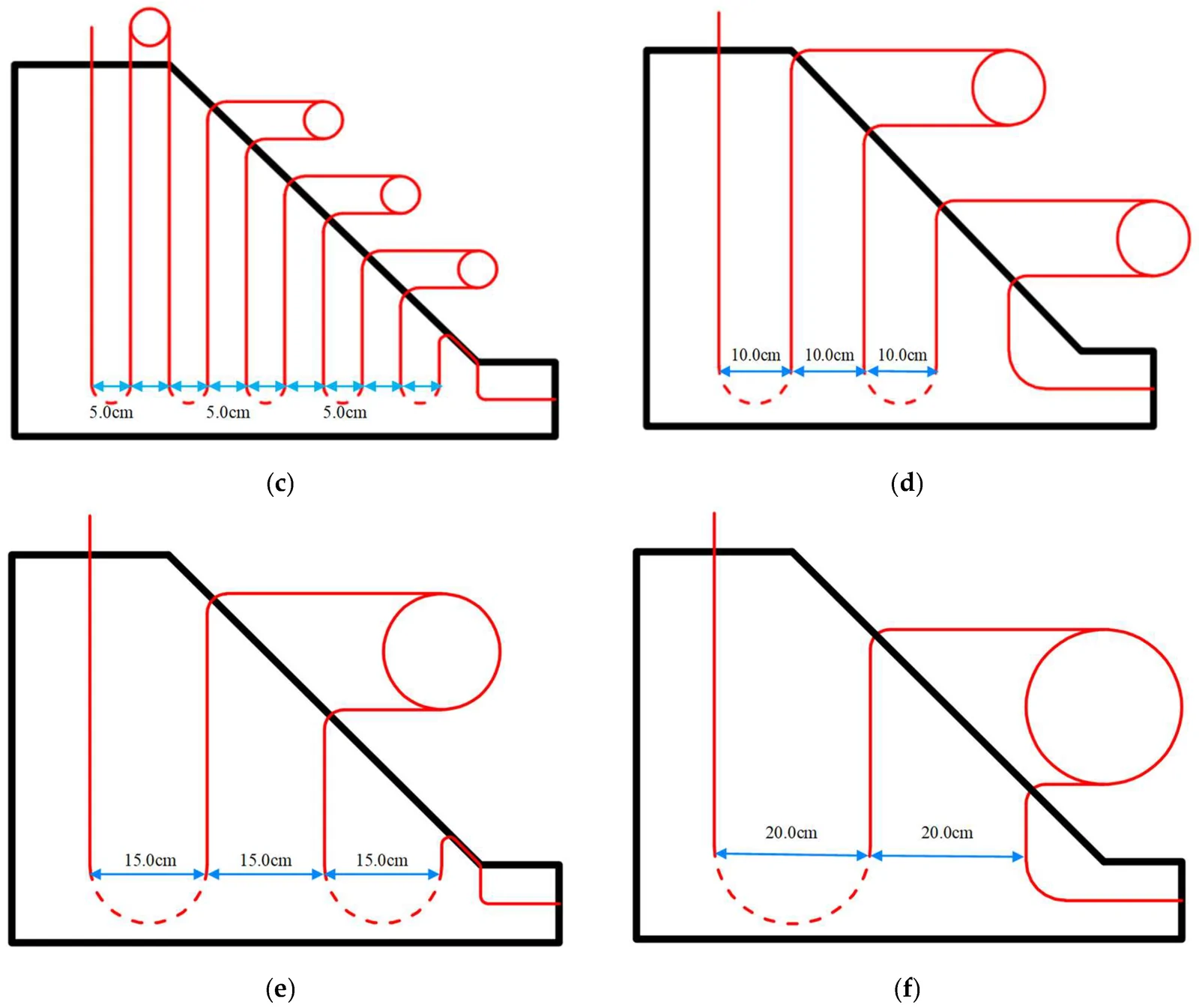
Schematic diagram of cable deployment at different spacings: (**a**) Test condition 1; (**b**) test condition 2; (**c**) test conditions 3 and 7; (**d**) borehole spacing of 10.0 cm; (**e**) borehole spacing of 15.0 cm; (**f**) borehole spacing of 20.0 cm.

The specific test conditions of this study are shown in Table 6. Under the Rainstorm condition, the strain-sensing optical fiber cables were deployed in vertical and horizontal boreholes to examine the influence of cable deployment direction. The boreholes were arranged at equal intervals with a spacing of 15 cm, as shown in Figure 10a,b.

For the moderate rain and light rain conditions, the strain-sensing optical fiber cables were deployed in vertical boreholes only. The boreholes were arranged at equal intervals with spacings of 5 cm, 10 cm, 15 cm, and 20 cm to examine the influence of borehole spacing. The strain-sensing optical fiber cables were anchored using heat-shrink tubes with a diameter of 5.0 mm and an anchoring spacing of 20 cm. The specific layouts are shown in Figure 10c–f.

In practical engineering, one sensing optical fiber cable is installed in each monitoring borehole. In the model test, to simplify the deployment procedure, a single cable was deployed by turning back inside the model. The turning positions are indicated by dashed lines in the figures for distinction.

The non-monitoring portions of the optical fiber cable consisted of a front lead-in section and intermediate lead sections. The front lead-in section included the patch cord and the initial cable segment before the cable entered the slope model, whereas the intermediate lead sections were the cable lengths reserved outside the slope when the cable was led out, turned, and introduced into the next borehole. Therefore, the distance recorded by the OFDR system represents the cumulative distance along the entire cable path rather than the horizontal coordinate of the physical model. To facilitate the interpretation of the strain distributions, the lengths of the intermediate lead sections were measured, as listed in Table 7. The same cable-path arrangement and intermediate lead lengths were adopted for the moderate- and light-rain tests with the corresponding borehole spacings.

### 3.4. Test Procedure

**Figure 11:**
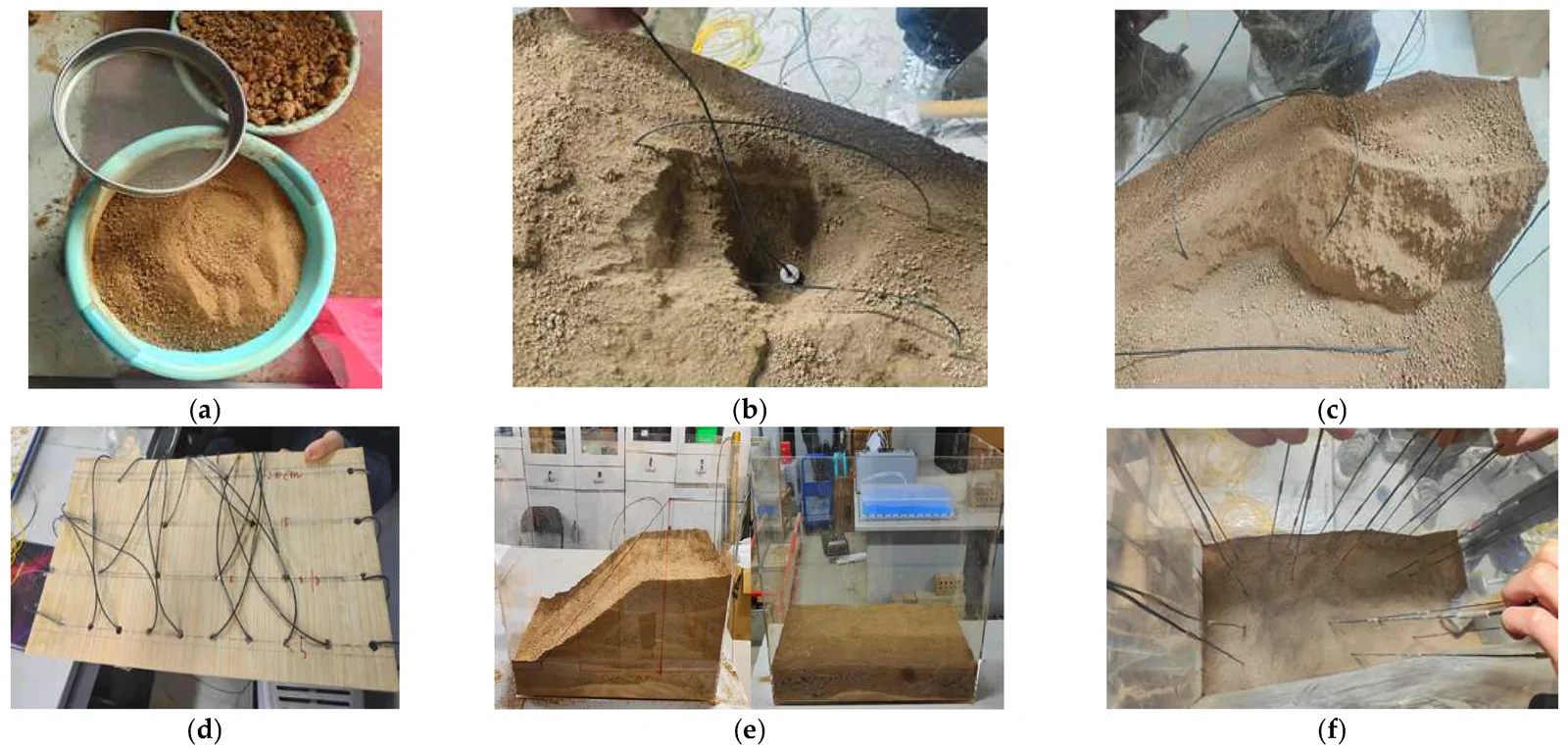
Photographs of the test process: (**a**) Sieving of test soil; (**b**) sensor embedding; (**c**) embedding of sensing optical fiber cables; (**d**) spacing control board; (**e**) layer-by-layer filling and compaction of the slope model; (**f**) cable deployment at different spacings.

The specific implementation procedure of the model test and the photographs records of the test process are shown in Figure 11.

(1) Sample preparation: The test soil was crushed and air-dried for two days, then passed through a 2 mm sieve with a small amount of 5 mm soil added. The soil was mixed uniformly using a mixer, and water was sprayed while mixing until the moisture content reached the preset moisture content.

(2) Soil filling: The weight of each layer (10 cm) of fill soil was calculated. The soil was poured into the model box layer by layer, and each layer was compacted with a wooden hammer to align the slope surface line with the marker line on the side of the model box. The slope angle was adjusted to 45° through this filling method. To facilitate the embedding of optical fiber cables in the light rain and moderate rain tests, a cable spacing control board was specially fabricated (see Figure 11d). First, the optical fiber cables at different spacings were threaded through the board and fixed with waterproof tape. The control board was then placed at a height of 5 cm from the bottom of the box. At this point, all optical fiber cables were kept vertical and slightly tensioned to avoid twisting or excessive bending. After the above preparation was completed, the fill soil was compacted layer by layer.

(3) Moisture conditioning: To improve the uniformity of moisture content within the slope model, the slope surface was fully covered with a transparent plastic sheet after slope construction, and the model was allowed to rest for 24 h at a room temperature of 20 ± 2 °C to ensure adequate moisture diffusion.

(4) Sensor embedding: After slope construction was completed, moisture sensors and earth pressure cells were embedded at depths of 7 cm and 5 cm below the slope surface, respectively, and the specific positions of each measurement point were marked. After the sensors were installed in place, their signal output terminals were connected to the data acquisition system via data cables, allowing real-time data acquisition by the earth pressure acquisition system and the moisture rapid tester to be carried out synchronously. Due to the large number of optical fiber cables under the moderate rain and light rain conditions, a lead-in section was reserved for each cable at the turning point where the cable extended to the outer edge of the slope, and the lead-in sections were fixed with adhesive tape on both inner sides of the test box to avoid confusion and enable precise positioning. The optical fiber cables were embedded layer by layer during the slope filling process. After all embedding was completed, the initial strain field of the slope was measured, recorded, and stored as the reference state for subsequent strain measurements.

(5) Rainfall and high-speed camera setup: The high-speed cameras were arranged, the water pump power was turned on, and the pump speed was adjusted to meet the corresponding rainfall intensity requirements. Water was added to the storage tank every 10 min to keep the water level in the tank constant, ensuring rainfall uniformity.

(6) Data monitoring: The strain-sensing optical fiber cable at the patch-cord end was connected to the OFDR demodulator. The axial strain distribution of the cable was recorded every 60 s, while soil moisture content, earth pressure, and slope surface morphology data were acquired simultaneously. During the rainfall process, the dynamic evolution of the slope surface under the scouring effect was continuously observed. The appearance time and development process of erosion gullies were recorded in detail, and key erosion features such as the time of collapse at the slope toe, slope surface, and slope crest were monitored. After the rainfall ended, the final slope surface morphology was recorded, with particular attention paid to failure modes such as cracks caused by the development of erosion gullies. All embedded sensors were monitored synchronously throughout the entire process. Rainfall continued until the slope showed obvious signs of sliding.

## 4. Results and Analysis

### 4.1. Wetting Front Analysis

The wetting front is an important physical quantity that characterizes moisture migration, and its dynamic variation is a decisive condition for the deformation of soil slopes. In this study, time was taken as the independent variable, and the rainfall infiltration characteristics were summarized by comparing the wetting fronts at different locations of the slope under different rainfall intensities.

**Figure 12:**
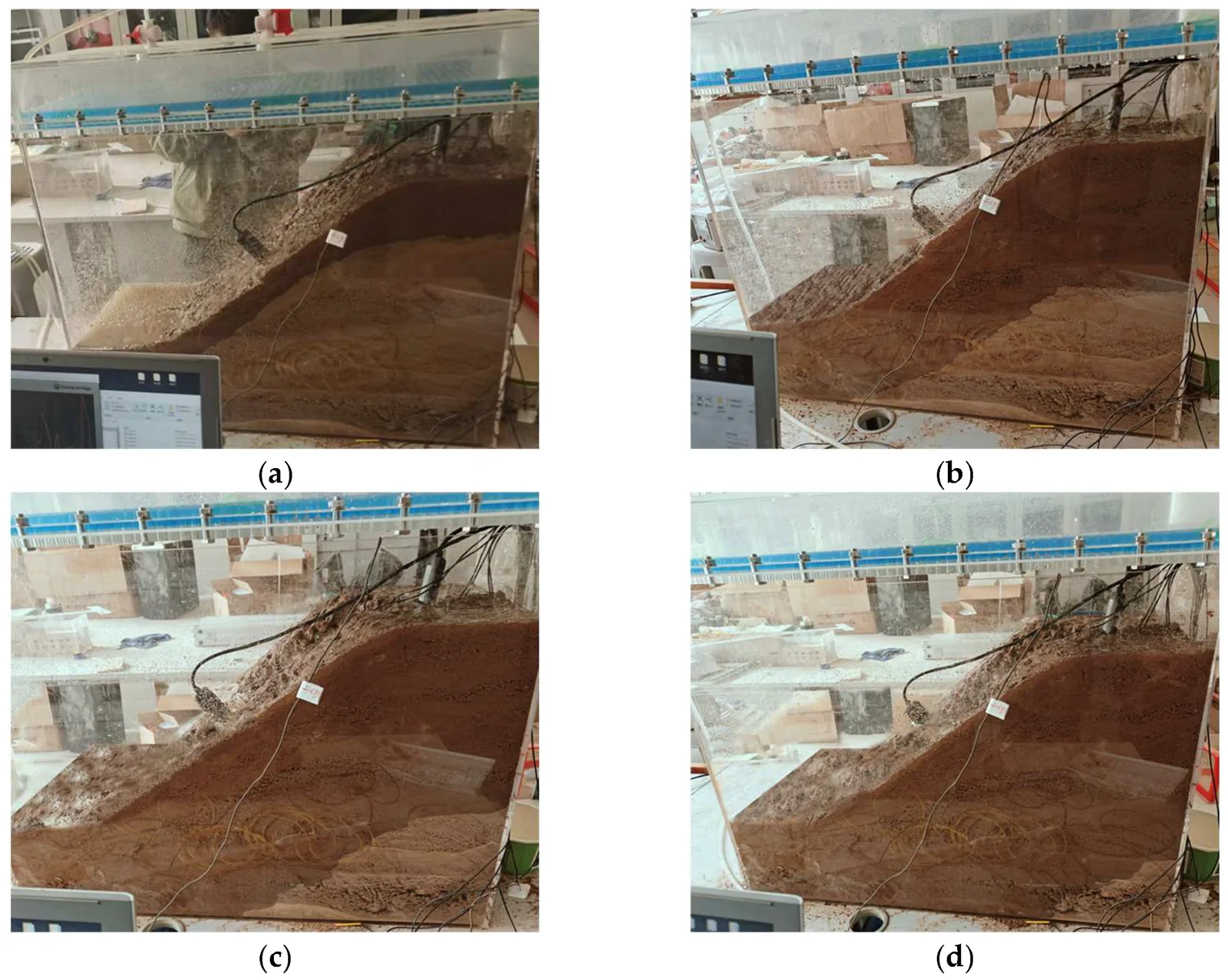
Spatial distribution of the wetting front in the slope at different times under the rainstorm condition: (**a**) 20 min; (**b**) 50 min; (**c**) 70 min; (**d**) 90 min.

**Figure 13:**
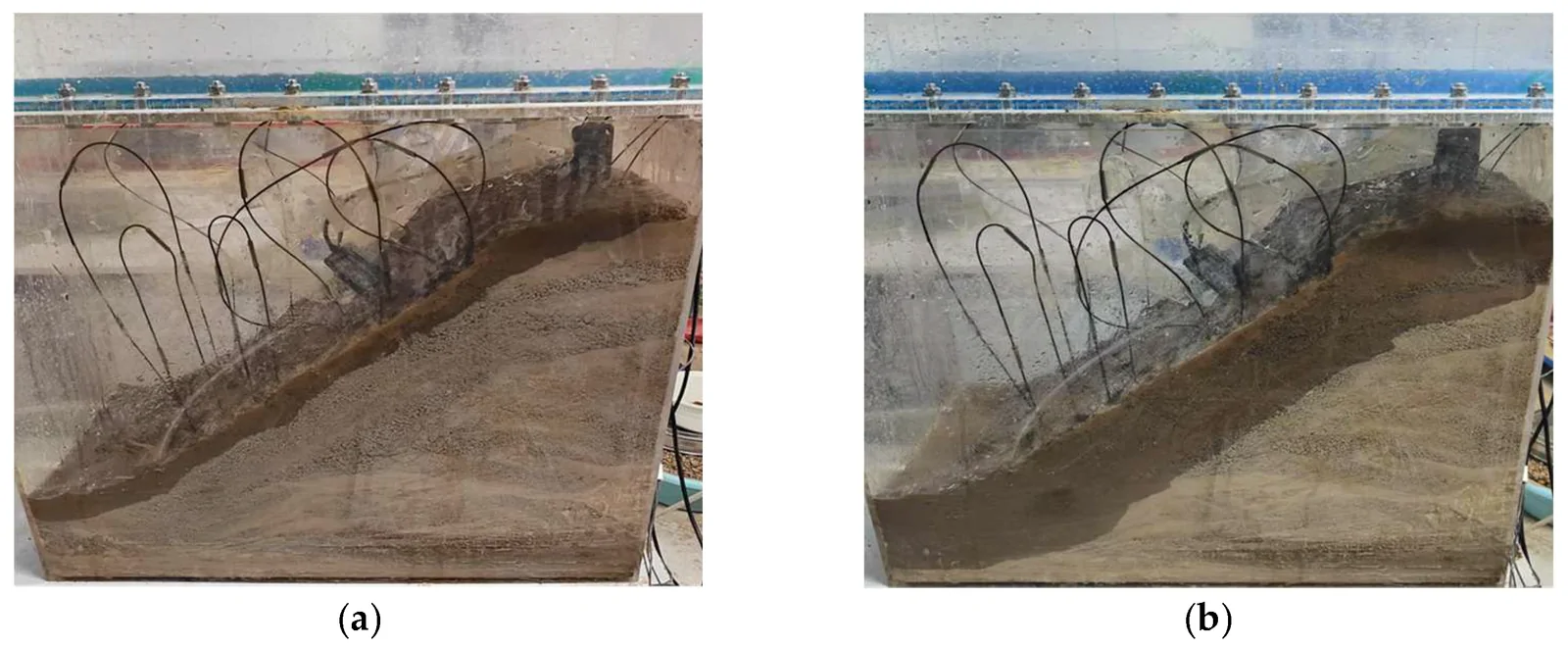

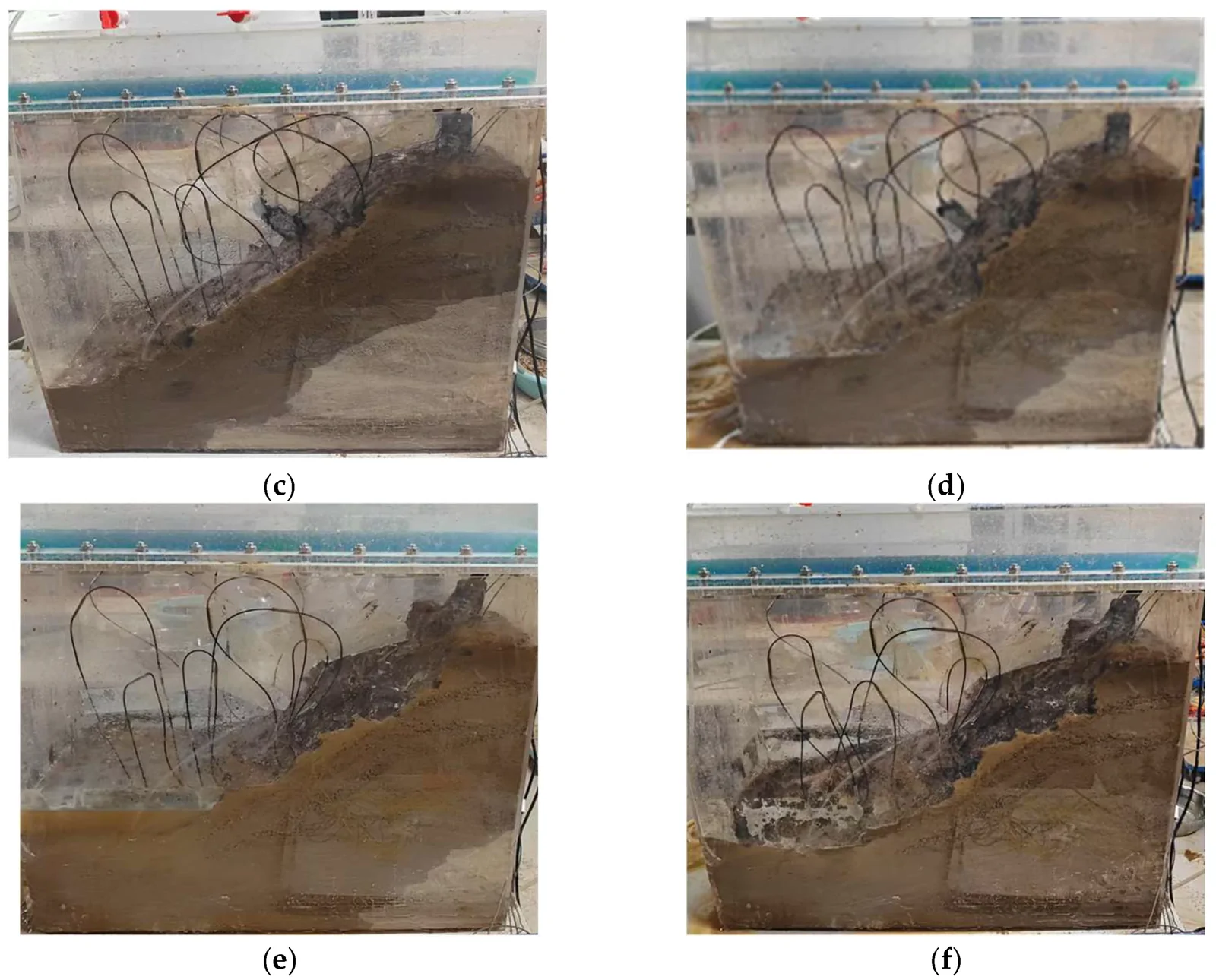
Spatial distribution of the wetting front in the slope at different times under the moderate rain condition: (**a**) 10 min; (**b**) 50 min; (**c**) 90 min; (**d**) 140 min; (**e**) 170 min; (**f**) 230 min.

**Figure 14:**
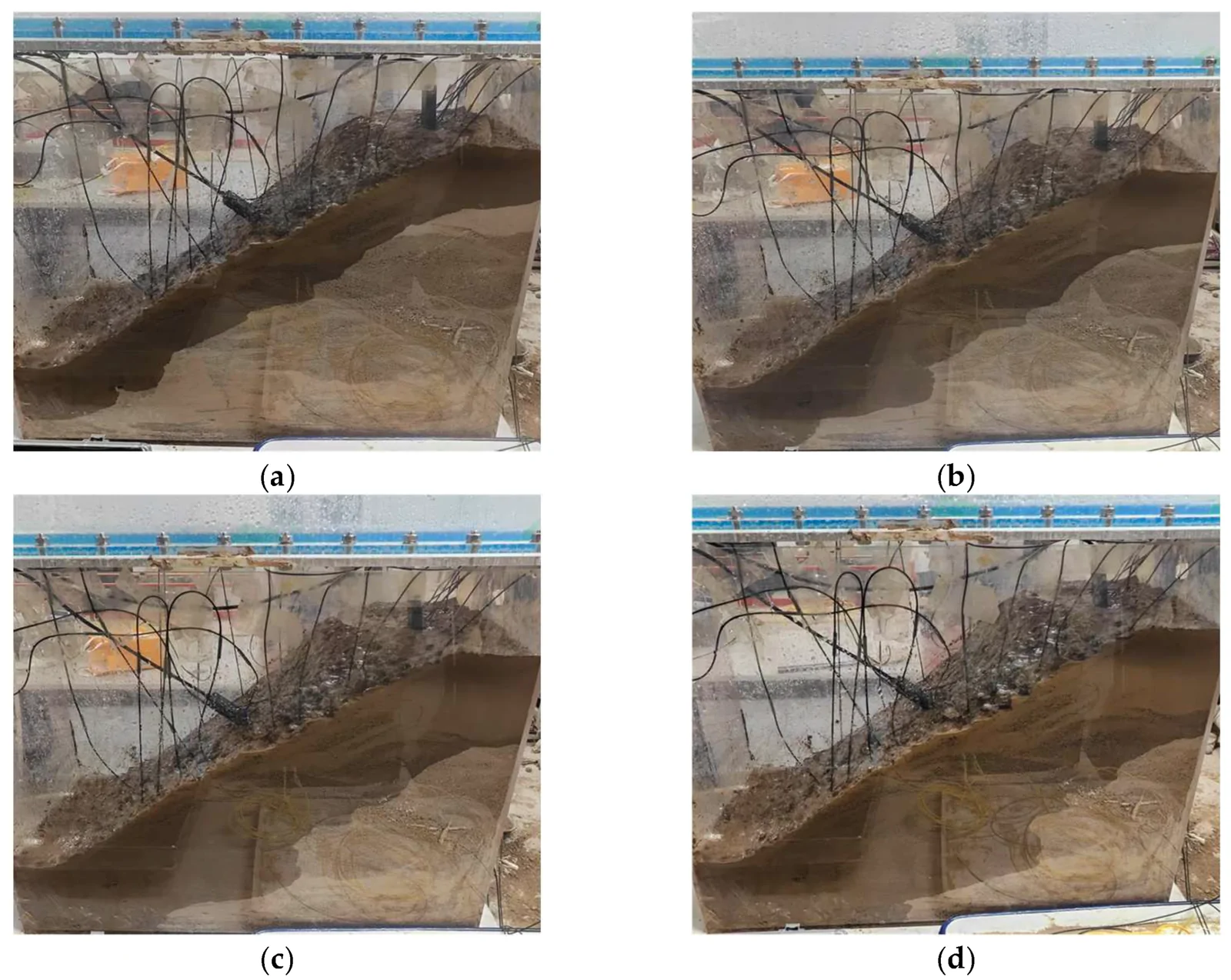

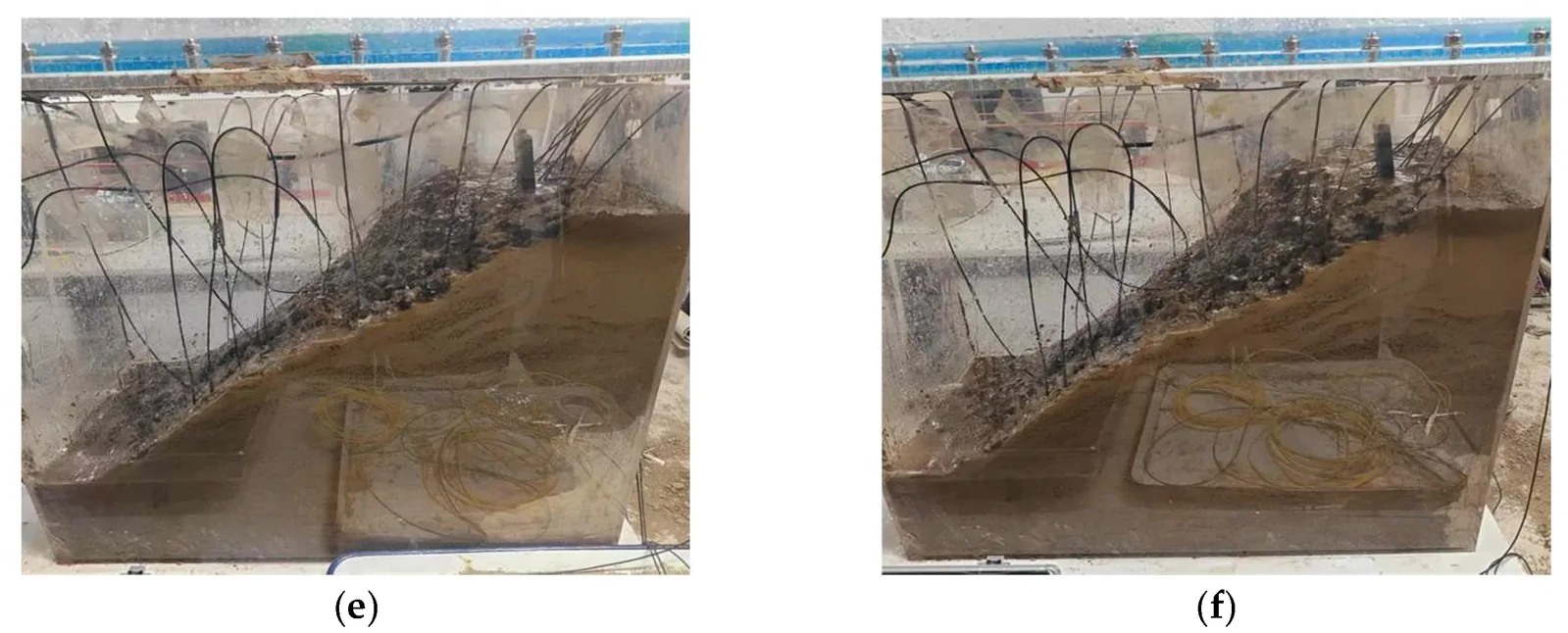
Spatial distribution of the wetting front in the slope at different times under the light rain condition: (**a**) 50 min; (**b**) 90 min; (**c**) 130 min; (**d**) 170 min; (**e**) 270 min; (**f**) 320 min.

Figure 12, Figure 13 and Figure 14 show the wetting front profiles when the slope was fully wetted under three rainfall intensity conditions, which intuitively reflect the influence of rainfall intensity on the water infiltration process. By comparing the wetting front height at 50 min and the time required for complete infiltration of the slope, the following observations were obtained:

Under the rainstorm condition, the wetting front height at 50 min was 25.63 cm, and the complete infiltration time was 100 min. Under the moderate rain condition, the wetting front height at 50 min was 11.80 cm, and the complete infiltration time was 230 min. Under the light rain condition, the wetting front height at 50 min was 8.89 cm, and the complete infiltration time was 320 min. These results indicate that a higher rainfall intensity significantly accelerates the water infiltration rate in the soil, promotes a faster downward migration of the wetting front, and thus shortens the time required for complete wetting of the slope. In addition, due to the lower slope height at the slope toe, the wetting front reached the bottom of the slope toe first under continuous rainfall, while the wetting front in the slope crest platform area had not yet reached the bottom. During this process, the soil within the slope was simultaneously subjected to the combined effects of downward moisture migration from the upper wetted zone and inward moisture migration from the slope toe wetted zone, until the entire slope soil became completely wetted.

### 4.2. Soil Moisture Content Analysis

**Figure 15:**
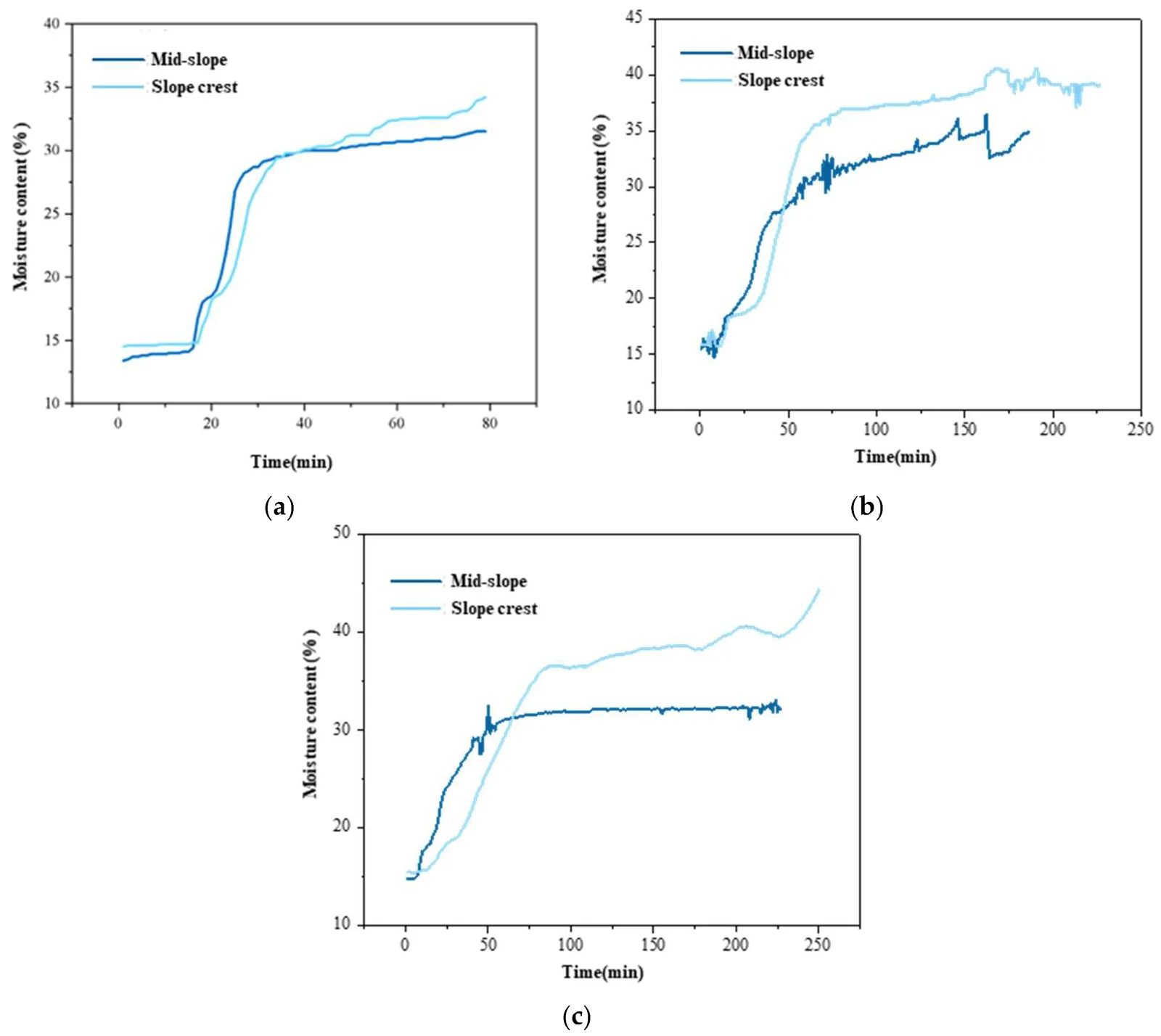
Variation curves of moisture content at different measurement points under different rainfall intensities: (**a**) rainstorm; (**b**) moderate rain; (**c**) light rain.

Figure 15 illustrates the variation in moisture content at the measurement points at the slope crest and the mid-slope under three rainfall intensity conditions. As shown in Figure 15, the moisture content at the slope crest was consistently higher than that at the mid-slope. This is mainly because the water on the slope surface primarily migrated downward and was readily drained, whereas the water flow in the slope crest area was relatively slow. In addition, due to the influence of local micro-topography, water tended to accumulate more easily in this area. As the rainfall intensity increased, the rising segment of the moisture content curve shifted notably earlier, indicating that the rainfall infiltration rate increased with increasing rainfall intensity. Under the moderate rain and light rain conditions, the moisture content monitoring data at the mid-slope were interrupted at 180 min and 230 min, respectively. At this time, slope surface runoff had caused significant scouring of the slope body, leading to the detachment of the surface soil. The scouring depth exceeded the embedding depth of the sensors, making the sensors unable to measure normally.

### 4.3. Earth Pressure Analysis

**Figure 16:**
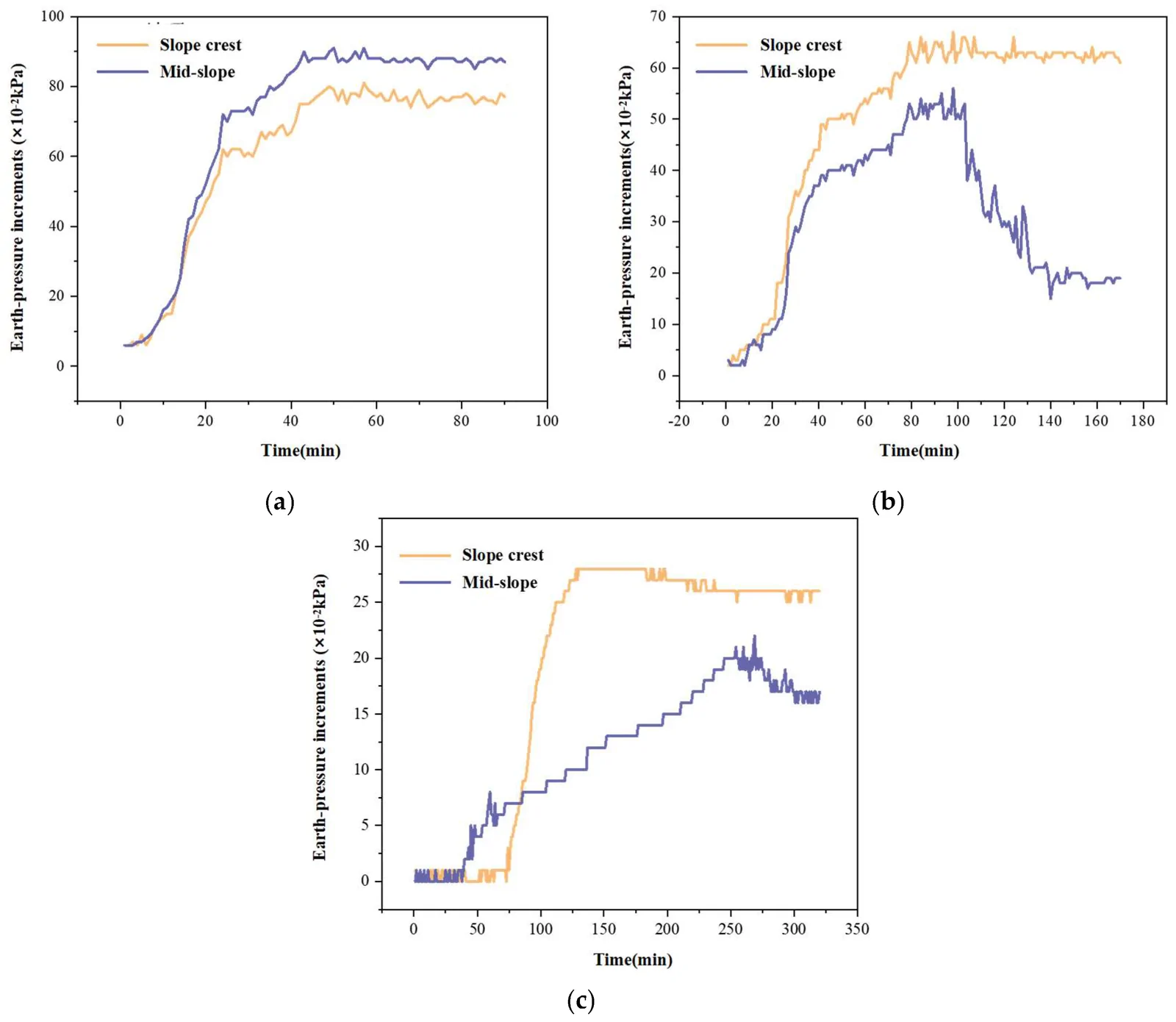
Variation curves of earth pressure recorded by EP1 at the slope crest and EP2 at mid-slope under different rainfall intensities: (**a**) Rainstorm; (**b**) moderate rain; (**c**) light rain.

Figure 16 illustrates the variation in rainfall-induced earth-pressure increments recorded by EP1 at the slope crest and EP2 at mid-slope under different rainfall intensity conditions, with the post-burial stable readings taken as the initial reference. As shown in Figure 16, the earth-pressure increment increased significantly with increasing rainfall intensity and showed an overall increasing trend during the rainfall process. This increase was attributed not only to the increase in soil unit weight caused by rainfall infiltration, but also to the reduction in matric suction and wetting-induced compression of the loess, which induced local stress redistribution around the sensors. However, under the light rain and moderate rain conditions, the earth-pressure increment at the slope surface showed a decreasing trend in the later stage of rainfall. The reason is that the slope surface soil was gradually eroded and detached under the scouring effect of surface runoff, reducing the thickness of the overlying soil above the sensors and promoting local stress release, thereby causing a corresponding decrease in the earth-pressure increment.

### 4.4. Measured Strain Analysis of Optical Fiber Cables

**Figure 17:**
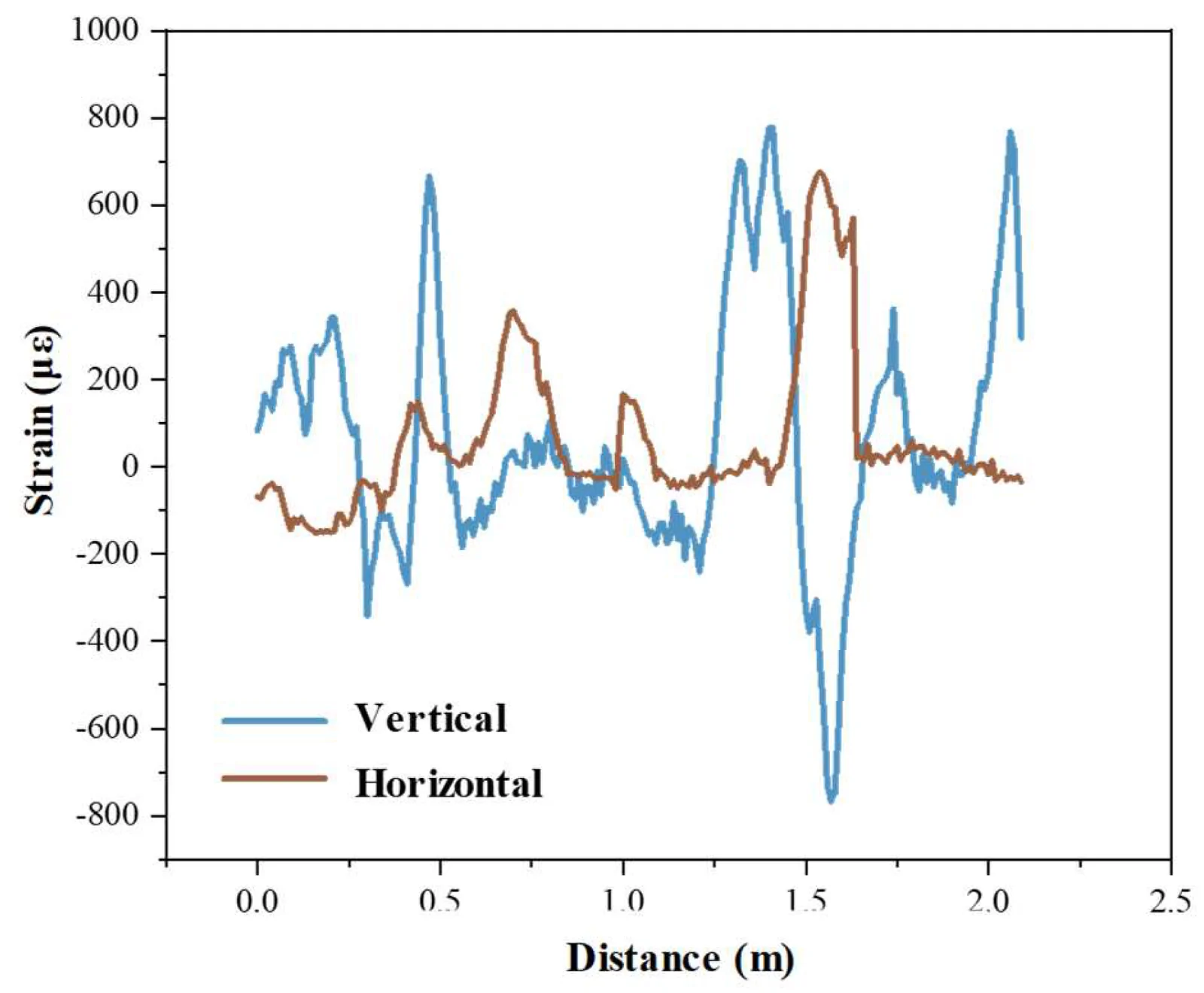
Spatial distribution of cable strain during rainfall-induced slope deformation under the rainstorm condition.

**Figure 18:**
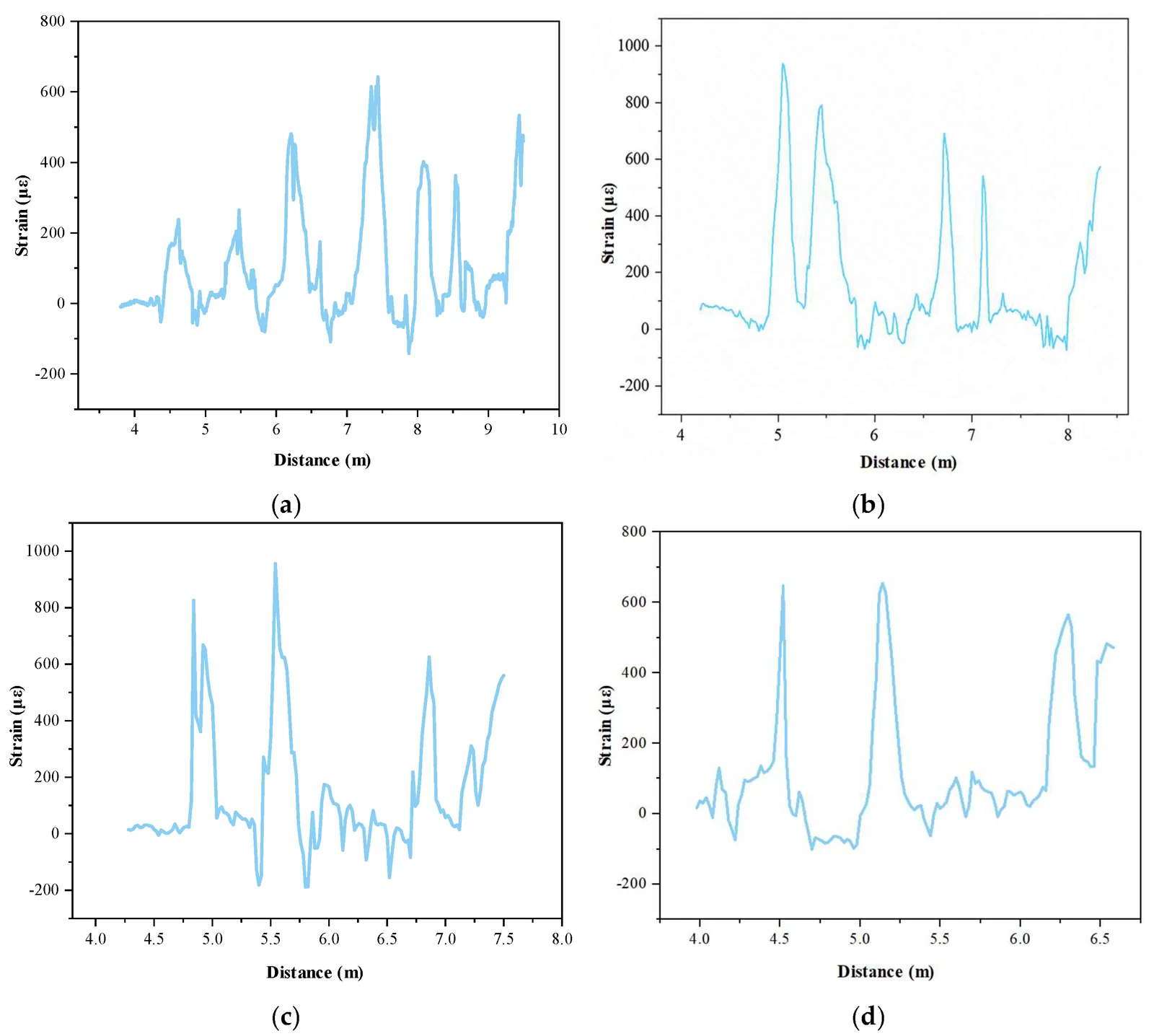
Measured strain distributions of cables at different borehole spacings during slope deformation under the moderate rain condition: (**a**) Borehole spacing of 5 cm; (**b**) borehole spacing of 10 cm; (**c**) borehole spacing of 15 cm; (**d**) borehole spacing of 20 cm.

**Figure 19:**
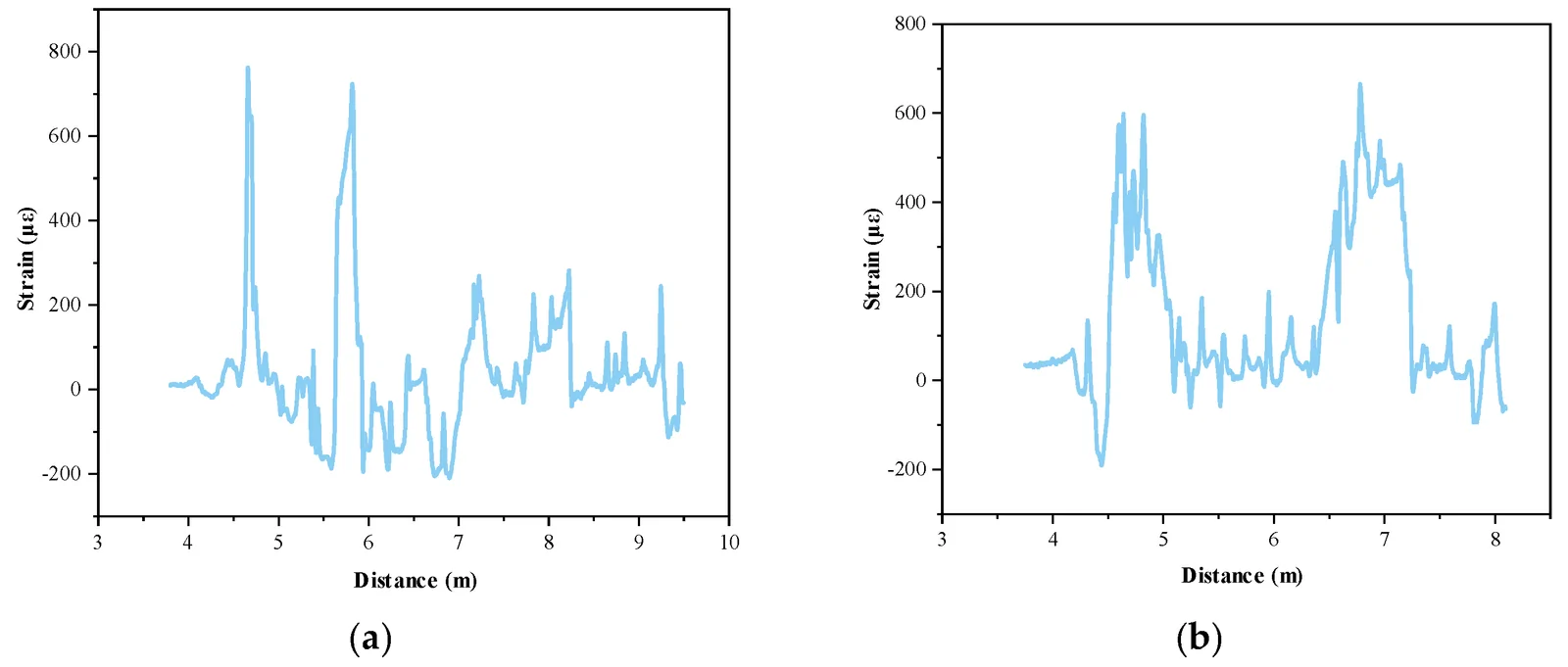

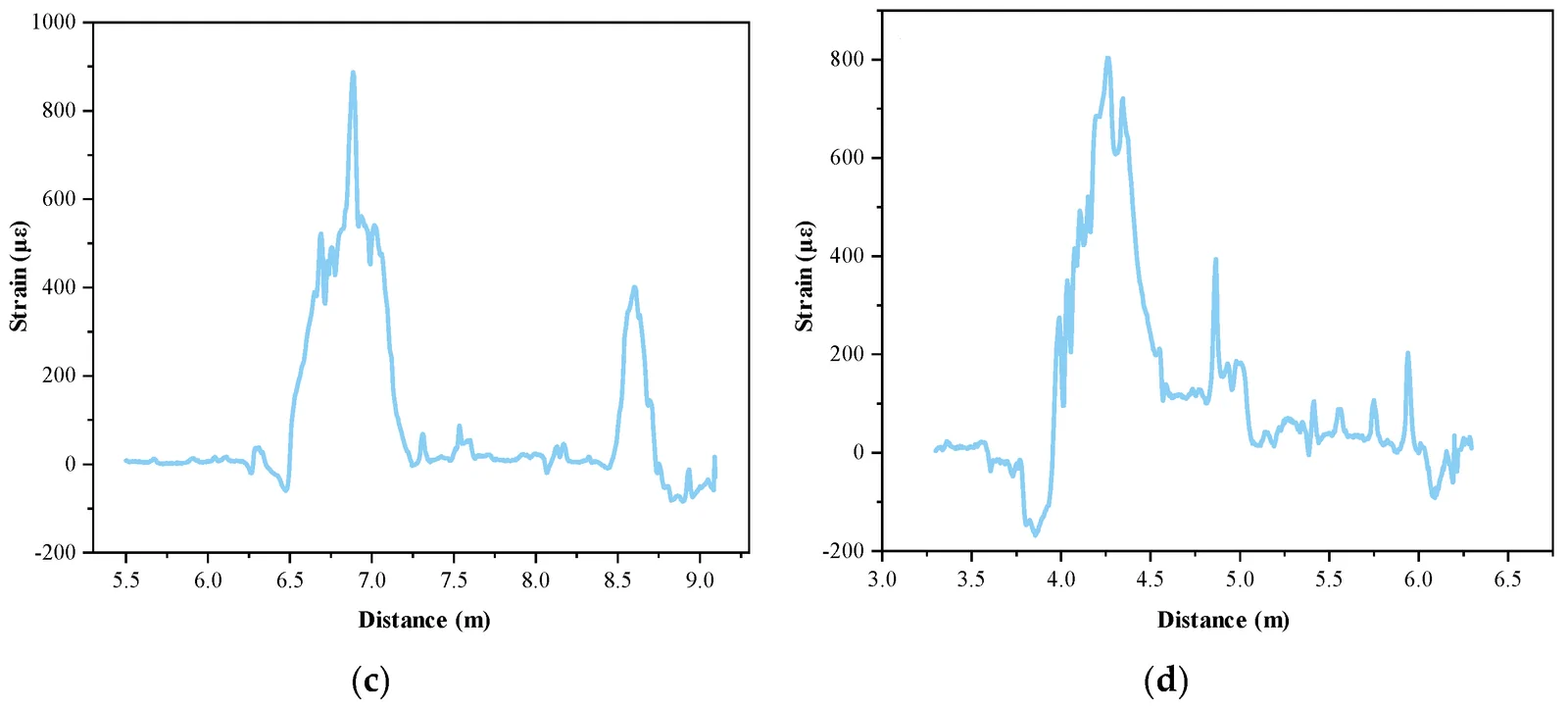
Measured strain distributions of cables at different borehole spacings under the light rain condition: (**a**) borehole spacing of 5 cm; (**b**) borehole spacing of 10 cm; (**c**) borehole spacing of 15 cm; (**d**) borehole spacing of 20 cm.

Figure 17, Figure 18 and Figure 19 present the measured strain distributions of the sensing optical fiber cables under different rainfall intensities and borehole spacings. For strain-data interpretation, only the effective cable sections embedded within the slope were considered, whereas the lead-in and turning sections were excluded. Strain anomaly points were identified as distinct local extrema relative to the adjacent measurements within each effective cable section. According to the predefined cable deployment path, the corresponding fiber-optic distances were converted into the burial positions of the cable sections within the slope. Overall, the cable strain did not vary smoothly and continuously along the monitoring path, but exhibited pronounced peaks at several specific locations, indicating that the internal deformation of the slope under rainfall was characterized by significant localized concentration. As rainfall intensity increased, the soil moisture content rose, and the matric suction and shear strength gradually decreased, allowing further development of internal slope deformation, which led to enhanced local tension, compression, or shear deformation of the cable. Therefore, the location and magnitude of the strain peaks can provide useful spatial indicators of localized internal deformation within the slope.

Under the rainstorm condition, as shown in Figure 17, the cable strain curves exhibited abrupt peak anomalies with relatively large local strain magnitudes, indicating that significant deformation concentration zones formed within the slope body during the rainfall-induced deformation process. These peak responses were generally consistent with the strain distribution characteristics of the cable under shear action, suggesting that when the slip surface or strong deformation zone intersected the cable, the cable was subjected to local shear or combined tension–compression effects, thereby producing pronounced strain anomalies. Therefore, the development position of the internal strain-concentration zone can be roughly identified based on the location of the strain peaks or strain anomaly points.

Comparing the different cable deployment methods in Figure 17, the vertically deployed cable exhibited two main strain peaks within the slope deformation influence range, whereas the horizontally deployed cable detected three distinct peaks. The larger number of peaks in the horizontal cable indicates that it can more continuously reflect the internal deformation distribution of deforming soil mass. This may be because the horizontal deployment method helps maintain a relatively uniform distribution of overburden pressure, thereby improving the coupling stability between the cable and the soil. In contrast, although the vertically deployed cable had fewer peaks, its peak anomaly characteristics were more pronounced, making it more effective in reflecting local deformation near the intersection with a strong deformation zone. Considering that in practical engineering, cables are mostly embedded by backfilling in vertical boreholes [45], the anchored cables were deployed vertically in both the moderate rain and light rain conditions in this test.

Figure 18 illustrates the influence of different borehole spacings on the strain characteristics of the anchored cables under the moderate rain condition. As the borehole spacing increased from 5 cm to 20 cm, the number of identifiable peaks in the cable strain curves generally decreased, indicating that increasing the borehole spacing reduces the spatial resolution of deformation identification. At a spacing of 5 cm, the strain peaks were relatively densely distributed, which could more fully reflect the localized deformation concentration zones within the slope deformation influence range. At spacings of 10 cm and 15 cm, the main peaks remained identifiable, but the local deformation information was somewhat attenuated. At a spacing of 20 cm, the monitoring results mainly reflected a few significant deformation zones, with reduced capability to identify small cracks and weak deformation zones. Under the moderate rain condition, 8, 5, 4, and 3 strain peaks were identified at spacings of 5 cm, 10 cm, 15 cm, and 20 cm, respectively, which corresponded well with the number of deployed optical fiber cables, indicating that the anchored distributed optical fiber system can effectively capture the internal deformation characteristics of rainfall-induced slope deformation. Within the tested range, reducing the borehole spacing increased the number and spatial density of identifiable strain anomalies, whereas increasing the spacing reduced the capability to resolve localized deformation.

Figure 19 presents the measured strain distributions of the cables at different borehole spacings under the light rain condition. Compared with the moderate rain and rainstorm conditions, although local peaks still appeared in the cable strain curves under light rain, the overall strain characteristics were weaker and did not exhibit the pronounced strain characteristics associated with accelerated slope deformation. This indicates that, under the test conditions, light rain infiltration did not produce evidence of obvious overall slope failure, and the overall slope remained in a relatively stable state. The local strain peaks may be related to localized collapsible settlement, minor settlement, soil structure adjustment, and early-stage deformation disturbances induced by rainfall infiltration, suggesting that the distributed optical fiber sensing system can capture weak deformations within the slope under light rain. However, slope instability generally develops progressively under the combined effects of rainfall infiltration, strength attenuation, deformation accumulation, and failure propagation. Although no obvious overall slope failure occurred under the light rain condition, the rainfall duration was the longest among the three conditions. Prolonged infiltration may lead to a gradual increase in the moisture content of the slope, thereby weakening the matric suction and shear strength of the loess and promoting the continuous accumulation of local deformation. Therefore, the absence of overall failure during the present test does not imply that the slope has no potential instability risk; rather, it indicates that the overall sliding failure condition had not yet been reached within the given test duration. If the rainfall duration were extended, the early-stage local deformation might continue to develop and gradually evolve into more extensive slope instability.

**Figure 20:**
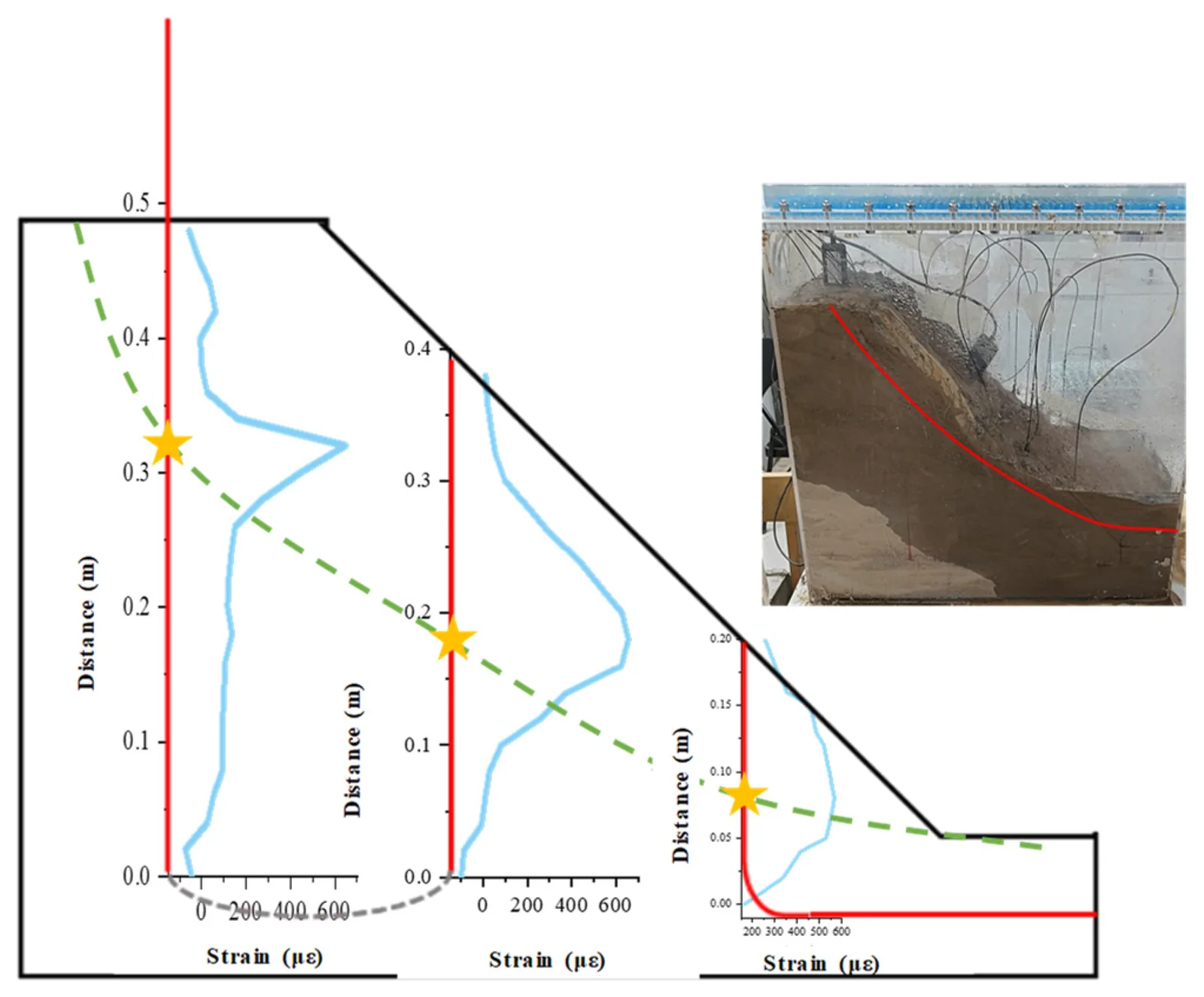
Internal strain-concentration zone within the slope characterized by distributed optical fiber strain monitoring.

Figure 20 shows the strain distribution measured by the optical fiber cable under the moderate rain condition with a borehole spacing of 20 cm. For clarity, only the strain distribution of the cable segments embedded inside the slope is shown. When visible localized deformation developed, the cable at the deformed zone exhibited a significant strain response due to shear action. Based on the strain-anomaly identification procedure described above, the anomaly points on the vertically embedded cable sections were connected by a smooth curve, providing an approximate representation of the internal strain-concentration zone. The three cables arranged from the slope crest to the slope toe detected strain peaks at positions of 0.32 m, 0.18 m, and 0.07 m, respectively, and these anomaly points are marked with star symbols in the figure. A comparison between the strain-peak-based deformation zone and the visibly deformed region showed qualitative spatial correspondence, indicating that distributed optical fiber strain monitoring can characterize internal strain-concentration zones within the slope.

Although the distributed optical fiber cable can capture the strain response during slope deformation and help identify internal strain-concentration zones, the early warning of accelerated slope deformation is often even more critical for landslide monitoring. By analyzing the variation process curves of strain at typical measurement points, a preliminary exploration of potential deformation precursors was conducted.

Guo et al. [46] analyzed the failure process of loess slopes under continuous rainfall. In the initial stage of rainfall, water rapidly infiltrated along the slope surface and the interface. Surface runoff developed on the slope, causing rainwater to accumulate rapidly at the slope toe and accelerating its saturation. The moisture content at the slope toe changed first. As the test progressed, small-scale collapse first occurred at the slope toe under unsaturated conditions. In the present test, a collapse phenomenon was also observed at the left slope toe. The slope toe of a loess slope is a stress concentration zone. When the stress conditions of the slope change, the plastic zone at the slope toe develops, eventually leading to slope failure. As the location where localized failure was first observed in the present test, the slope toe requires particular attention.

**Figure 21:**
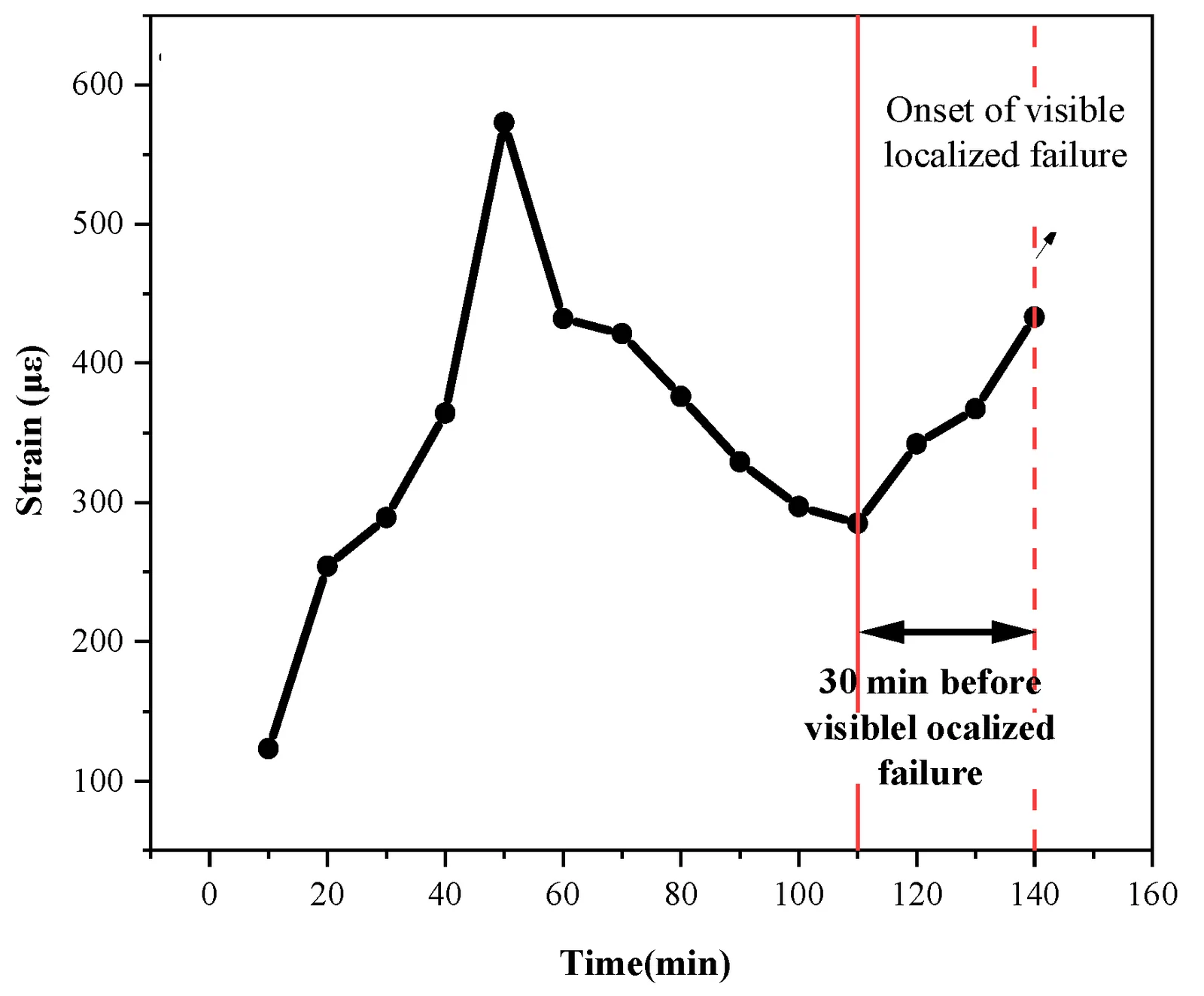
Variation of strain with time at 5 cm below the slope toe under the moderate rain condition.

Figure 21 shows the variation in strain with time at a measurement point located 5 cm below the slope toe under the moderate rain condition. It can be seen from Figure 21 that the strain at the slope toe reached a peak 50 min after the start of rainfall. Combined with the observations of wetting front development and slope surface morphology, the moisture content in the slope toe region had approached saturation at this time, but slope surface scouring was not yet significant, and the runoff had not caused substantial soil loss. The continuous infiltration of water increased the self-weight of the soil, and the overburden pressure on the cable increased correspondingly, leading to a rise in strain.

As rainfall continued to 90 min, local collapse occurred at the left slope toe, forming a free surface. The overlying soil on the cable was reduced, and the strain correspondingly decreased. At 120 min, the slope surface morphology indicated intensifying downslope deformation. By 140 min, the cable strain had increased to 433 με. Based on the slope surface morphology, substantial localized deformation was considered to have developed at this point.

Notably, the strain did not exhibit a sudden jump before the onset of visible localized failure, but instead gradually increased from 285 με to 433 με within the preceding 30 min. This progressive increase reflects the accumulation of deformation at the slope toe and may provide a potential precursor of accelerated slope deformation under the present test condition.

### 4.5. Slope Surface Morphology Analysis

**Figure 22:**
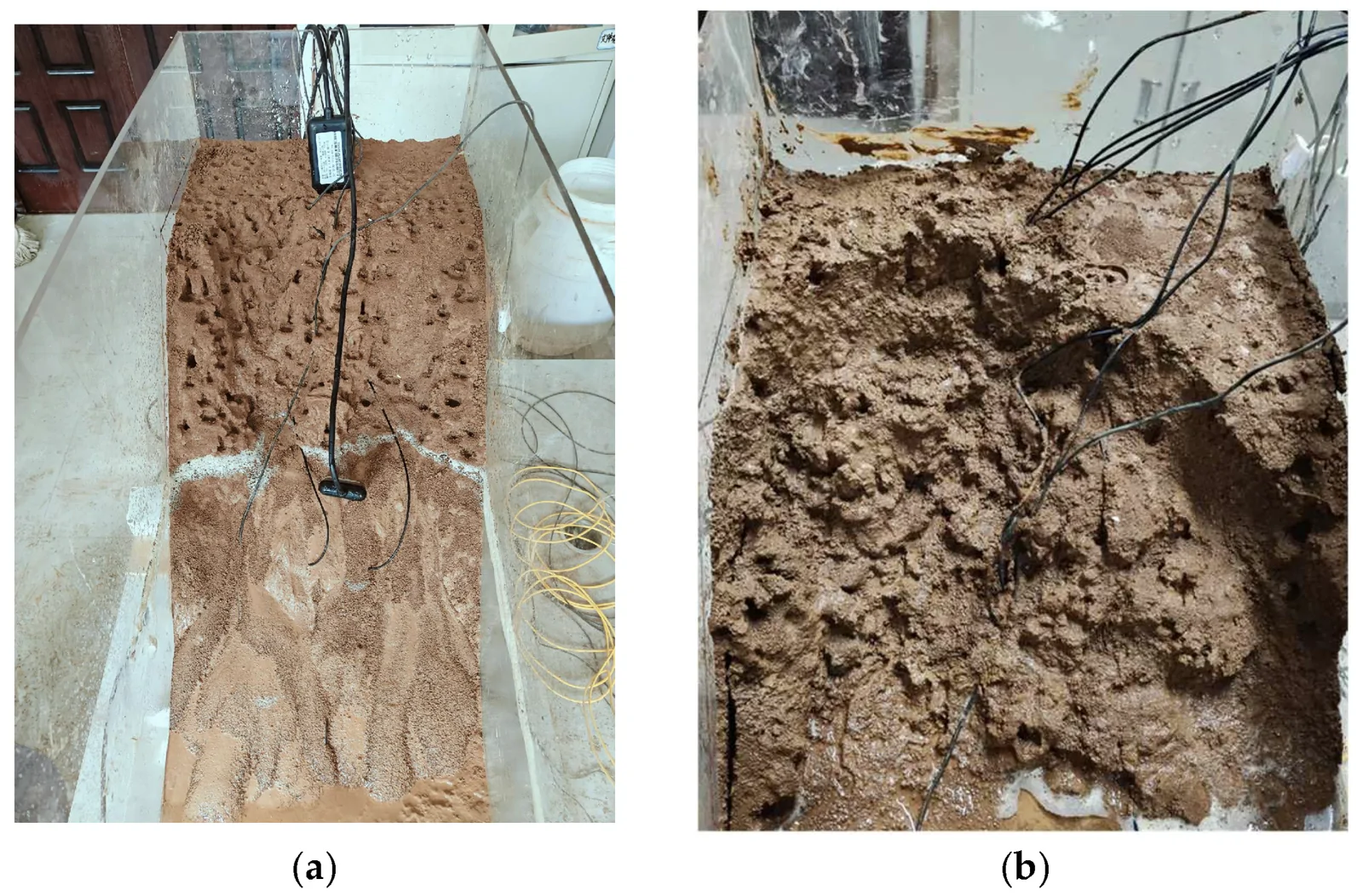
Slope surface erosion and shallow flow-like deformation under the rainstorm condition: (**a**) 90 min; (**b**) 130 min.

As shown in Figure 22, under the rainstorm condition, no obvious overall slope movement was observed when the wetting front reached the bottom of the slope at 90 min, and the entire slope surface remained stable. However, as rainfall scouring continued, numerous splash erosion pits appeared on the slope surface, and a shallow rill developed on the left side of the slope. Rainwater on the slope surface transported part of the soil along this rill. At 130 min of rainfall, the soil mass near the slope crest platform was observed to undergo shallow, flow-like downslope deformation along the eroded slope surface.

**Figure 23:**
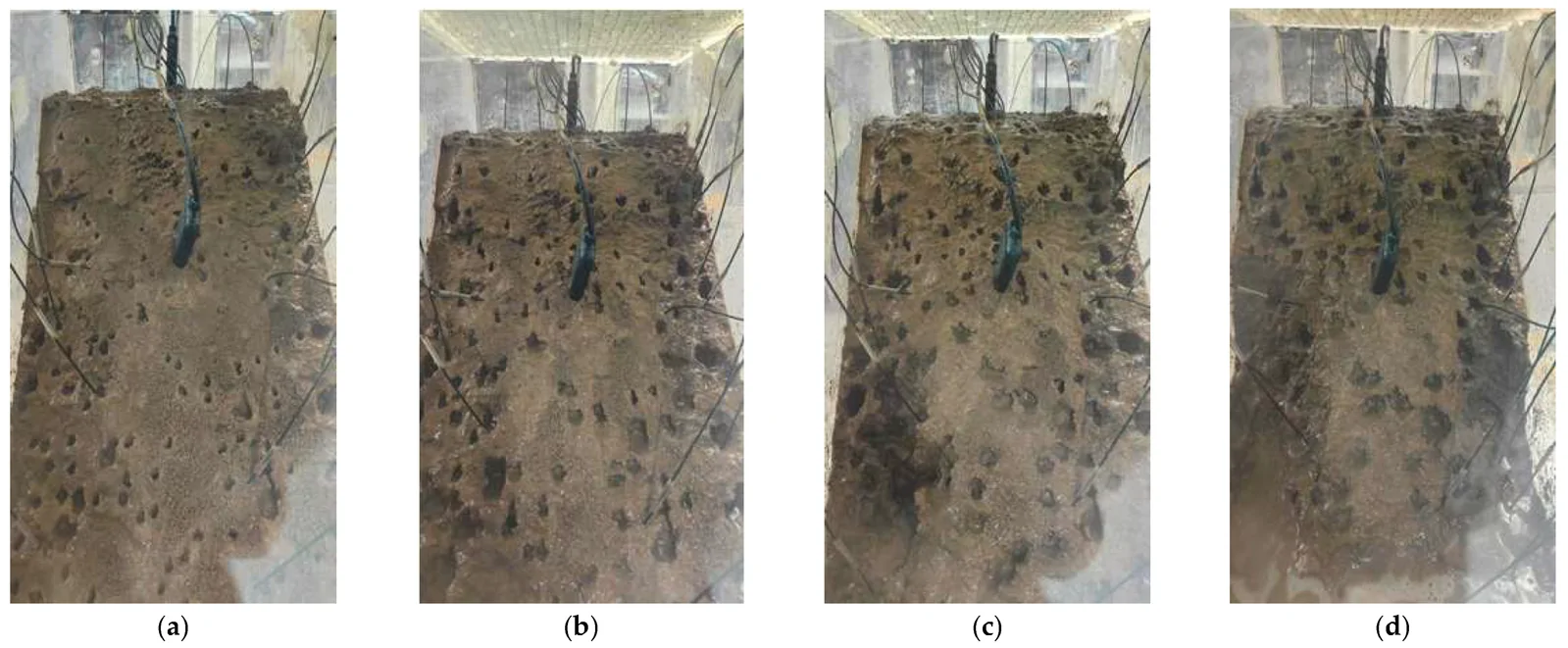

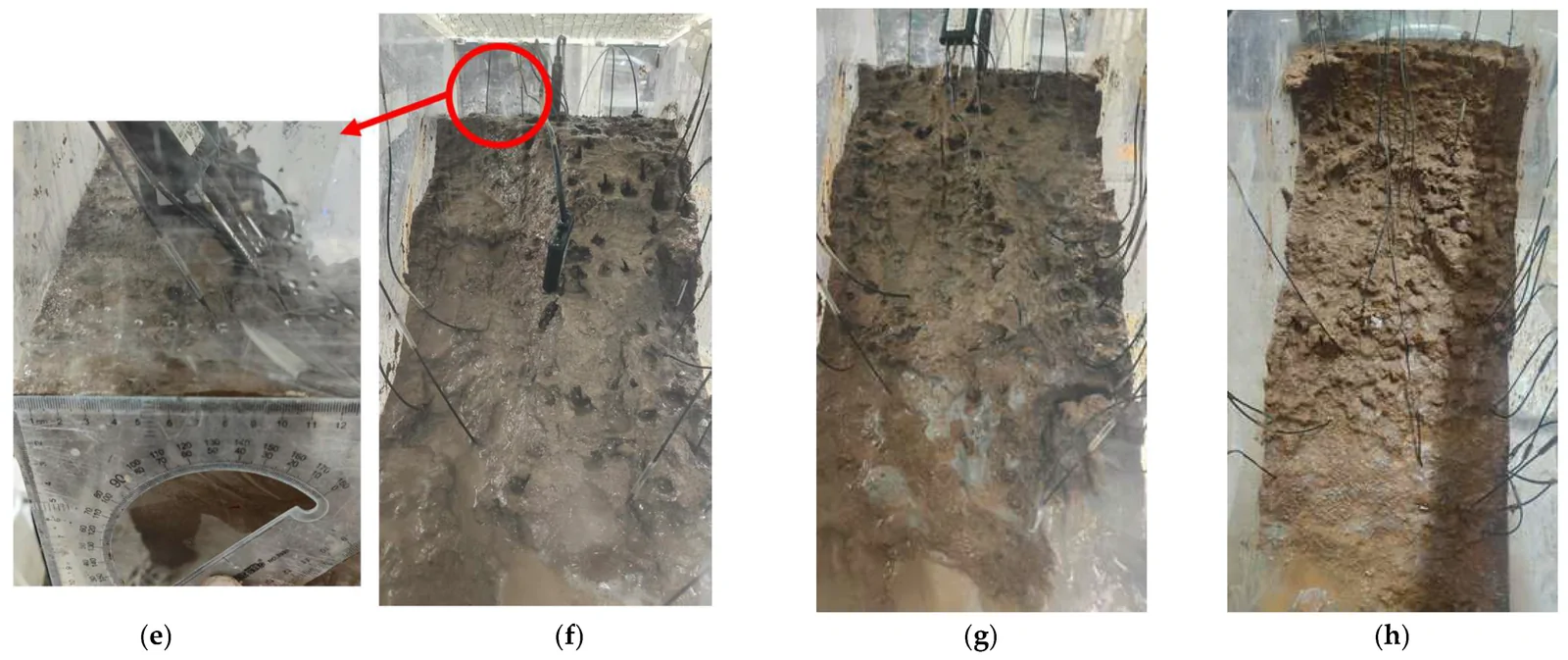
Slope surface erosion and progressive localized deformation under the moderate rain condition: (**a**) 20 min; (**b**) 50 min; (**c**) 90 min; (**d**) 120 min; (**e**) slope crest platform at 140 min; (**f**) 140 min; (**g**) 190 min; (**h**) final deformation morphology.

As shown in Figure 23, under the moderate rain condition, soil exfoliation first occurred at the left slope toe. Under the scouring effect of surface runoff, the amount of exfoliated soil increased, forming a large cavity at the left slope toe (Figure 23d). Subsequently, an the loss of toe support induced localized collapse and progressive deformation toward the upper slope (Figure 23e). Following this progressive deformation, the length of the slope crest platform decreased from 20 cm to 8 cm (Figure 23g). Then, soil exfoliation also appeared at the right slope toe, and the slope surface was severely damaged (Figure 23h).

**Figure 24:**
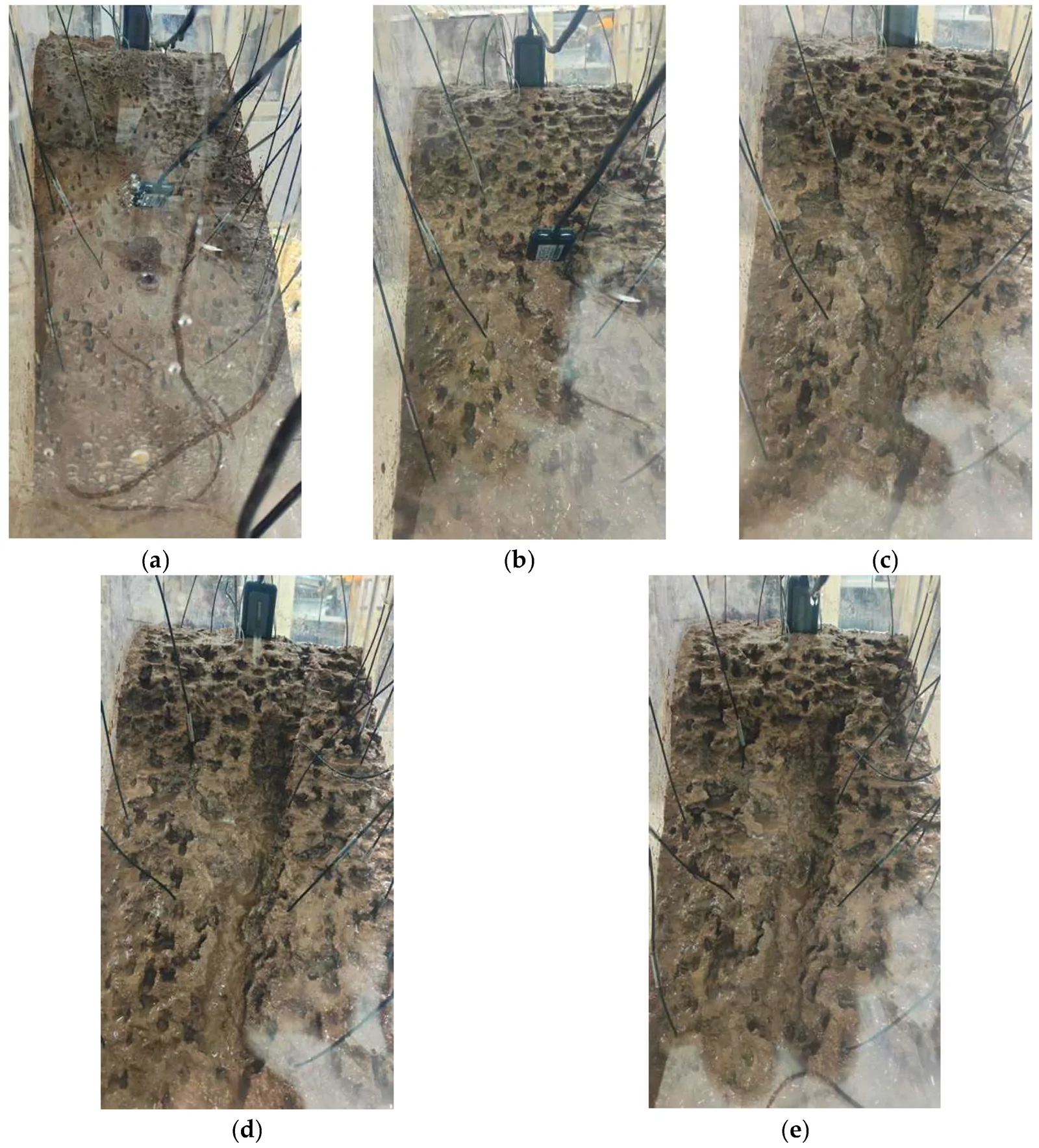
Slope surface erosion process under the light rain condition: (**a**) 20 min; (**b**) 210 min; (**c**) 230 min; (**d**) 260 min; (**e**) 320 min.

The slope surface erosion process under the light rain condition is shown in Figure 24. An erosion gully developed in the lower part of the slope, which continuously deepened, widened, and extended downstream as rainfall progressed. The surface runoff formed by rainfall transported the surface soil particles along the erosion gully. However, under the light rain condition, the rainfall duration was long and rainwater infiltration was sufficient, but no obvious overall slope failure was observed.

Overall, the observed failure process was characterized by surface erosion, toe scouring, localized collapse, and shallow flow-like deformation, reflecting the progressive development of rainfall-induced slope deformation. These observations were obtained from a remolded homogeneous loess slope with a specific geometry. Further studies under heterogeneous or layered soil conditions, different slope geometries, and field-scale conditions are needed to assess the broader applicability of the observed monitoring responses and deployment findings and to further clarify the geometry and continuity of the internal deformation boundary.

## 5. Conclusions

To apply distributed optical fiber strain sensing (DOFSS) to the monitoring of rainfall-induced landslide deformation in soil slopes, a deployment scheme and embedding technique for landslide-prone soil slope monitoring were proposed based on existing engineering experience and evaluated through laboratory-scale model tests. The main conclusions are as follows:

(1) For internal deformation monitoring of landslide-prone soil slopes, the vertical borehole deployment method is recommended. Tight-buffered cables with a diameter of 2 mm anchored with 5.0 mm heat-shrink tubes produced identifiable strain responses under the present laboratory test conditions. The deployment range of the strain-sensing optical fiber cables should be determined based on the potential sliding surface location obtained from geological investigation data and numerical simulation. The boreholes for cable deployment should cover the front, middle, and rear sections of the potentially unstable slope zone. The borehole depth should extend 1–2 m below the potential sliding surface. Borehole spacing has a significant influence on monitoring performance. Considering both monitoring accuracy and cost, a borehole spacing controlled within 20–48% of the potentially unstable slope zone is recommended.
(2) The laboratory model test results showed that the strain peaks measured by the optical fiber cables during progressive slope deformation were spatially associated with the visibly deformed regions. Connecting the strain-anomaly points identified along the vertically embedded cable sections provided an approximate representation of the internal strain-concentration zone. Through precise positioning of the cable, connecting the strain anomaly points provided an approximate indication of the internal strain-concentration zone. This indicates that distributed fiber-optic strain monitoring technology can characterize strain concentration zones within slopes.
(3) The variation patterns of monitoring data such as volumetric water content, earth pressure, and wetting front at different locations within the soil slope are highly correlated with the rainfall infiltration process and the evolution characteristics of surface erosion. These parameters can also serve as important indicators for assessing slope deformation and stability. Further field applications, large-scale model tests, and numerical parametric studies are needed to evaluate the applicability of the proposed deployment approach under different geological and engineering conditions and to explore the integration of multi-parameter monitoring data for slope-deformation assessment.

## Figures and Tables

**Table 1:** Statistics of borehole parameters for optical fiber cable embedding in selected landslide projects in China and abroad.

Landslide Name	Landslide Extent (m)	Number of Boreholes	Maximum Borehole Spacing (m)	Ratio of Max. Borehole Spacing to Horizontal Length of Landslide
Majiagou landslide [32]	560	3	200	0.36
Outang landslide [33]	1083	6	335	0.31
Xinpu landslide [34,35]	1900	1	-	-
Yuhuangge landslide [27]	450	2	217	0.48
Majiagou landslide [28]	460	6	117	0.25
A reinforced soil slope [36]	25	5	5	0.20
Gradenbach landslide [36]	1800	1	-	-
A road cut slope [37]	32	3	11	0.34
A subgrade slope in Qingdao [38]	11	5	1.90	0.17
A reinforced slope in Chongqing [39]	80	3	16	0.20

**Table 2:** Applicability comparison of different distributed optical fiber sensing technologies.

Technology	Sensing Principle	Spatial Resolution	Monitoring Distance	Applicable Scenarios
OTDR	Rayleigh scattering/Optical loss	Meter-level	Long distance	Long-distance distributed disturbance, optical loss variation, and macro-scale deformation response monitoring
BOTDR	Brillouin scattering	Meter-level	Long distance	Engineering slope monitoring in complex construction conditions, where the distal end of the cable is inaccessible, or the monitoring distance is long
BOTDA	Brillouin scattering	Meter-level to sub-meter-level	Long distance	Engineering monitoring where both ends of the cable are accessible and high accuracy of distributed strain measurement is required
OFDR	Rayleigh scattering	Millimeter-level	Short distance	Laboratory model tests and high spatial resolution strain monitoring in localized key areas

**Table 3:** Similarity ratios for the scaled laboratory slope model.

Physical Quantity	Similarity Ratio	Value
Cohesion (*c*)	$C_{c}$	1
Internal friction angle (*φ*)	$C_{\varphi }$	1
Displacement (*d*)	$C_{d}=C_{l}$	50
Time (*t*)	$C_{t}=C_{l}^{1/2}C_{g}^{-1/2}$	7.07
Poisson’s ratio (*υ*)	$C_{v}$	1
Strain (*ε*)	$C_{\varepsilon }$	1
Stress (*σ*)	$C_{\sigma }=C_{l}C_{\rho }C_{g}$	50
Shear modulus (*G*)	$C_{G}$	1
Elastic modulus (*E*)	$C_{E}$	1

**Table 4:** Basic physical parameters of the loess.

Natural Density (g·cm^−3^)	Initial Moisture Content (%)	Dry Density (g·cm^−3^)	Specific Gravity	Plasticity Index
1.71	12.80	1.60	2.74	16.90

**Table 5:** Classification criteria for rainfall grades.

Grade	12 h Rainfall (mm)	Test Setting (mm)
Light rain	0.10–4.90	2.50
Moderate rain	5.00–14.90	10.00
Rainstorm	30.00–69.90	50.00

**Table 6:** Test conditions.

Test Condition No.	Rainfall Intensity Grade	Cable Deployment Direction	Borehole Spacing (cm)	Cable Anchoring Method
1	Rainstorm	Horizontal	15.0	No anchoring
2	Rainstorm	Vertical	15.0	
3	Light rain	Vertical	5.0	Anchored with heat-shrink tubes of 5.0 mm diameter, anchoring spacing of 20 cm
4	Light rain	Vertical	10.0	
5	Light rain	Vertical	15.0	
6	Light rain	Vertical	20.0	
7	Moderate rain	Vertical	5.0	
8	Moderate rain	Vertical	10.0	
9	Moderate rain	Vertical	15.0	
10	Moderate rain	Vertical	20.0	

**Table 7:** Lengths of the intermediate lead sections for different borehole spacings.

Borehole Spacing (cm)	Intermediate Lead Lengths from the Slope Crest to the Slope Toe (cm)
5.0	44, 71, 51, 49
10.0	77, 76
15.0	78
20.0	82

## Data Availability

The data presented in this study are available from the corresponding authors upon reasonable request.
